# Supplementary material for: Drug-induced change in transmitter identity is a shared mechanism generating cognitive deficits
Source: Nat Commun. 2024 Sep 26;15:8260. doi: 10.1038/s41467-024-52451-x (PMC11427679; doi:10.1038/s41467-024-52451-x)
Supplement: Supplementary file 1 — Supplementary Information [file 41467_2024_52451_MOESM1_ESM.pdf]

Drug-induced change in transmitter identity is a shared mechanism generating cognitive deficits

Marta Pratelli [1,2]*, Anna M. Hakimi[1,2], Arth Thaker[1,2], Hyeonseok Jang[1], Hui-quan Li[1,2], Swetha K. Godavarthi[1,2], Byung Kook Lim[1], Nicholas C. Spitzer[1,2]*

Corresponding authors' e-mail addresses:

Marta Pratelli (mpratelli@ucsd.edu)

Nicholas C. Spitzer (nspitzer@ucsd.edu)

Supplementary Information

(Contains Supplementary Figures 1-18 with Legends and Supplementary Tables 1-24 with legends)

Supplementary Information

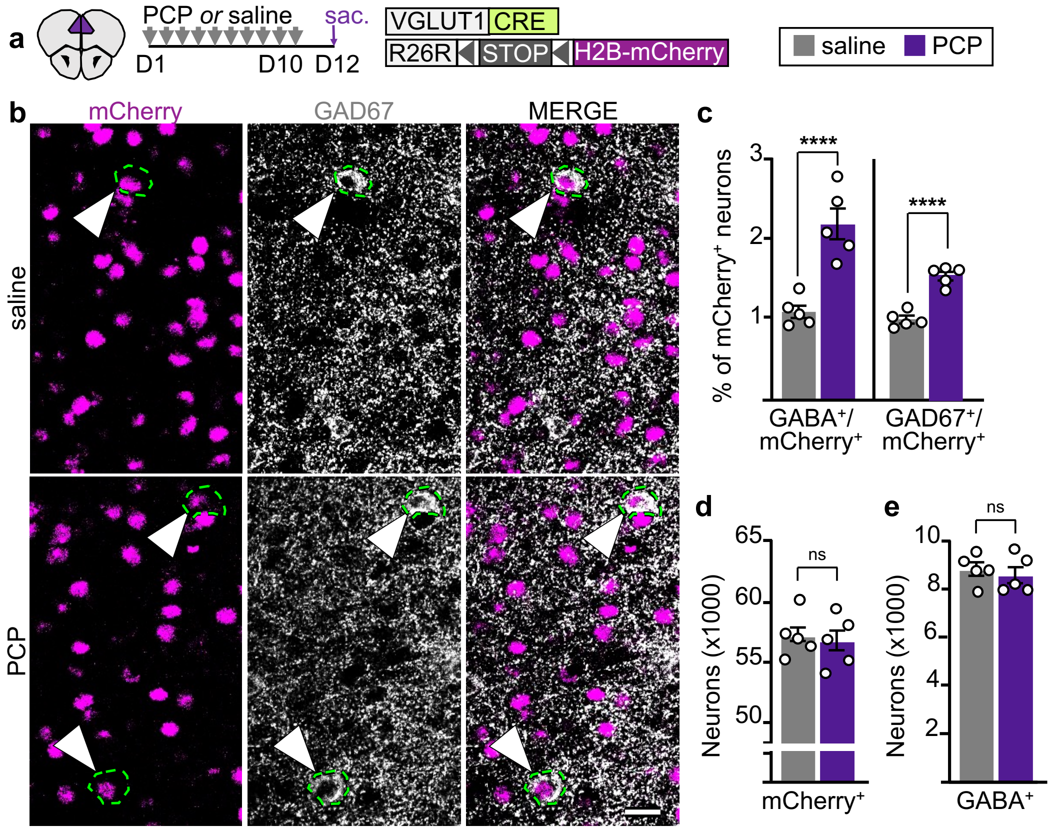

Supplementary Fig. 1 | PCP treatment increases the number of mCherry$^+$ neurons co-expressing GABA and GAD67 in the PL, but not the total number of mCherry$^+$ or GABA-only neurons. a Experimental protocol to investigate the effect of PCP treatment on the transmitter phenotype of PL neurons. **b** PL neurons co-expressing mCherry and GAD67 (arrowheads). Scale bar, 20 µm. **c** Percent of GABA$^+$/mCherry$^+$ and GAD67$^+$/mCherry$^+$ cells in total mCherry$^+$ neurons (*n*=5 mice). **d, e** Quantification of total mCherry$^+$ neurons and GABA$^+$/mCherry$^-$ neurons in the PL of PCP- and saline-treated mice (*n*=5 mice). Statistical significance (****P<0.0001) was assessed using two-sided unpaired t-test. Data are presented as mean ± SEM. The exact p-values and additional statistical details can be found in Supplementary table 8.

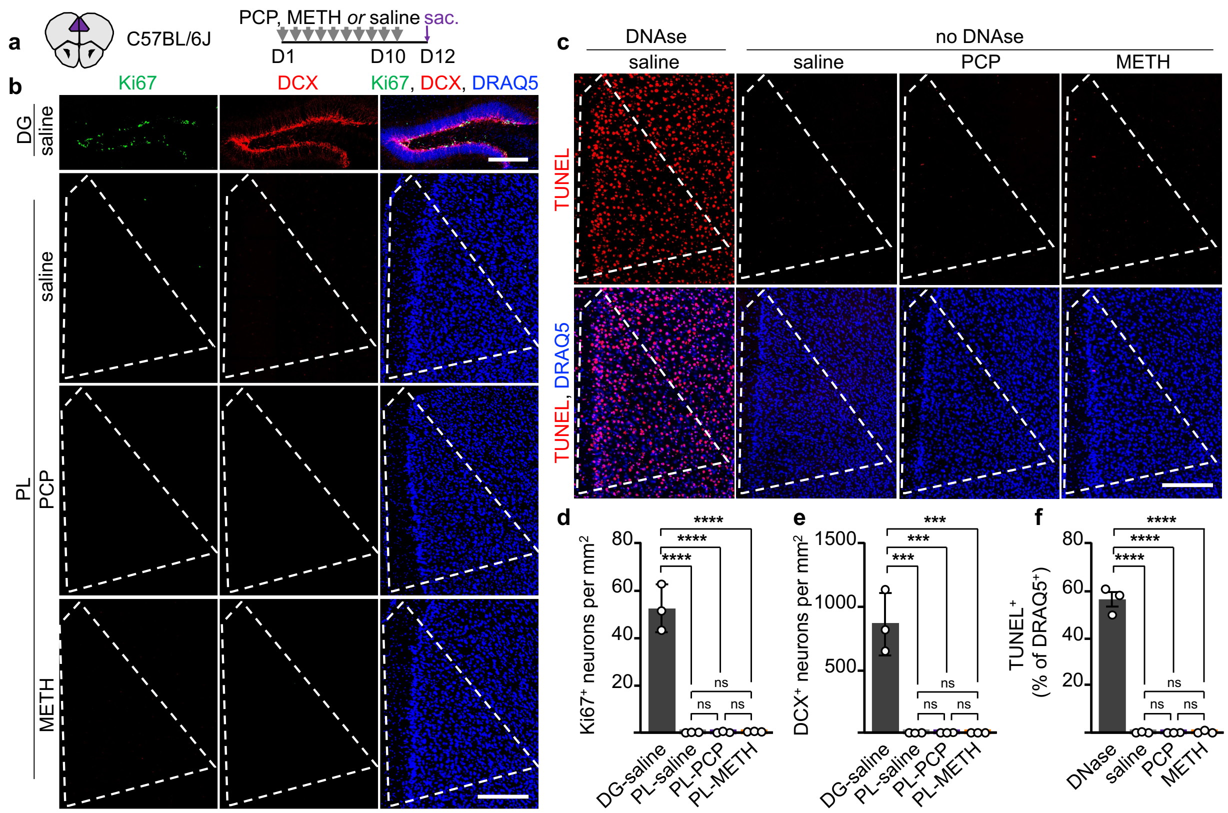

Supplementary Fig. 2 | Neurogenesis and apoptosis are not detected in the PL of PCP- or METH-treated mice. a Experimental protocol to evaluate whether drug-treatment causes apoptosis or neurogenesis in the PL. **b** Ki67, DCX, and DRAQ5 (nuclear staining) expression in the dentate gyrus (DG; upper panels) and PL (lower panels) of mice treated for 10 days with PCP, METH, or saline. Scale bar, 300 µm. **c** PL sections from PCP-, METH- or saline-treated mice double-stained for TUNEL and DRAQ5. Left panels contain a DNase-treated control section. Scale bar, 300 µm. **d**,**e** Quantification of $Ki67^+$ and DCX^+ cells in the DG and PL (*n*=3 mice). **f** Quantification of TUNEL signal in the PL of mice treated with PCP, METH, or saline (*n*=3 mice). Statistical significance (***P 0.001; ****P<0.0001) was assessed using one-way ANOVA with Tukey's multiple-comparisons test. Data are presented as mean ± SEM. The exact p-values and additional statistical details can be found in Supplementary table 9.

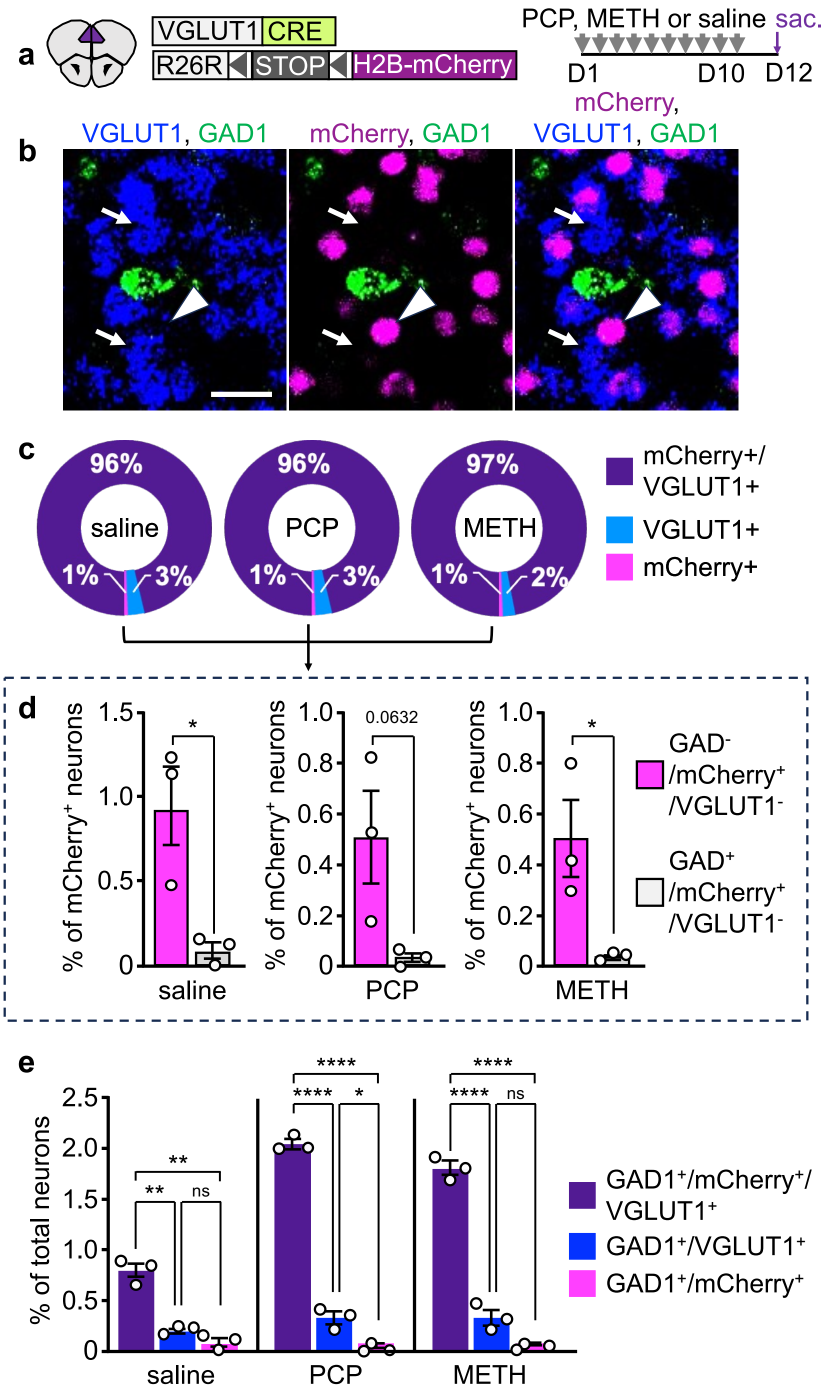

Supplementary Fig. 3 | Nonspecific mCherry labelling causes little co-expression of mCherry and GABA in the PL of VGLUT1^{CRE}::mCherry mice. a Experimental protocol to determine the efficiency and specificity of the VGLUT1^{CRE}::mCherry mouse line in labelling VGLUT1^{+} PL neurons, as well as the percent of glutamatergic neurons co-expressing or gaining GAD1. **b** Expression of VGLUT1, mCherry and GAD1 in the PL of a VGLUT1^{CRE}::mCherry mouse treated with saline. The arrowhead shows a mCherry^{+}/VGLUT1^{-} neuron and arrows indicate neurons that are VGLUT1^{+} but mCherry^{-}. Scale bar, 20 μm. **c** Percent expression of mCherry^{+}/VGLUT1^{+} neurons VGLUT1-only neurons, and mCherry-only neurons in the PL of VGLUT1^{CRE}::mCherry mice treated with PCP, METH or saline (*n*=3 mice). **d** Percent of mCherry^{+} neurons that are negative for VGLUT1 and do or do not express GAD1 across treatment groups (*n*=3 mice). **e**

Percent of neurons in which GAD1 is co-expressed with both VGLUT1 and mCherry, VGLUT1-only, or mCherry-only across treatment groups (*n*=3 mice). Statistical significance (*P 0.05; **P 0.01; ****P<0.0001) was assessed using two-sided unpaired t-test (**d**) or one-way ANOVA with Tukey's multiple-comparisons test (**e**). Data are presented as mean ± SEM. The exact p-values and additional statistical details can be found in Supplementary table 10.

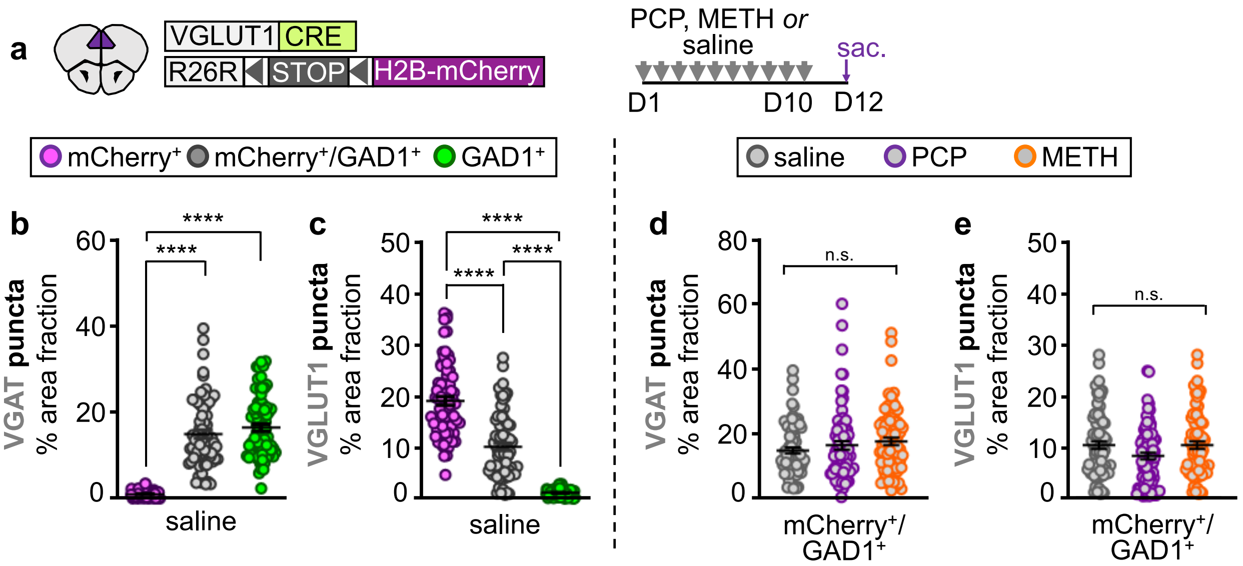

Supplementary Fig. 4 | The expression levels of VGLUT1 and VGAT in PL neurons of saline-treated controls resemble those observed in drug-treated mice. a Experimental protocol. **b**,**c** Quantification of expression level of VGAT (**b**) and VGLUT1 (**c**) in mCherry-only, GAD1$^+$/mCherry$^+$ and GAD1-only neurons in PL neurons of saline-treated controls (*n*=25 cells/type/mouse for 3 mice). **d**,**e** Comparison across treatment groups of the expression level of VGAT (**d**) and VGLUT1 (**e**) in PL GAD1$^+$/mCherry$^+$ neurons (n=25 cells/mouse for 3 mice/treatment). Statistical significance (****P<0.0001) was assessed using Kruskal-Wallis followed by Dunn's test. Data are presented as mean ± SEM. The exact p-values and additional statistical details can be found in Supplementary table 11.

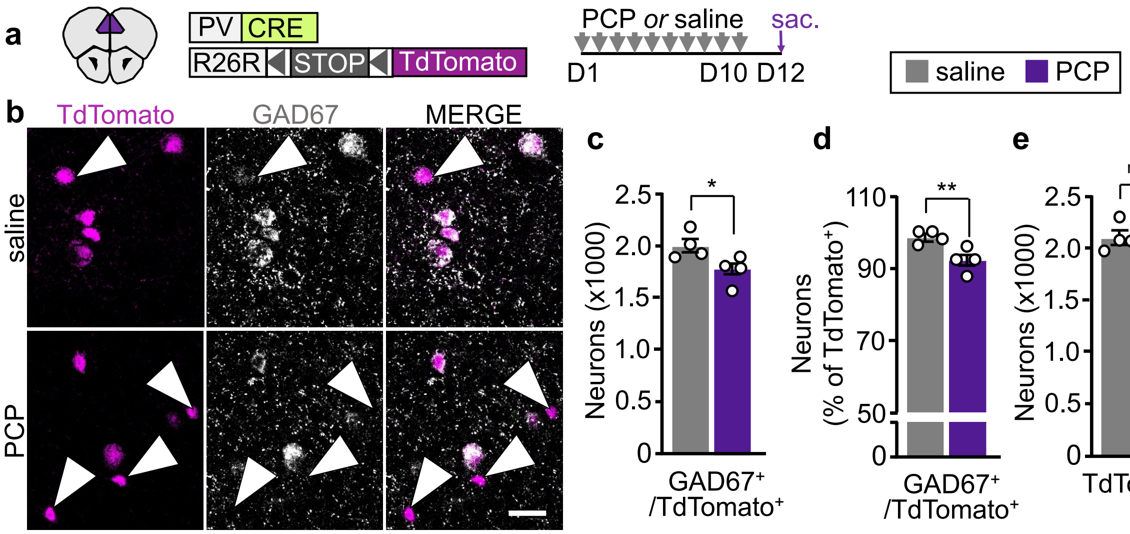

Supplementary Fig. 5 | Treatment with PCP reduces the number of PL PV+ neurons expressing GAD67. a Experimental protocol to assess GAD67 expression in PV neurons. **b** PL TdTomato+ labeling of PV+ interneurons, and GAD67+ cells across treatment groups. Arrowheads show TdTomato+ neurons lacking GAD67 expression. **c** Quantification of TdTomato+/GAD67+ neurons in the PL of PCP- and saline-treated mice (*n*=4 mice). **d** Percent of TdTomato+/GAD67+ cells in total TdTomato+ neurons (*n*=4 mice). **e** Quantification of TdTomato+ neurons in the PL of PCP- and saline-treated mice (*n*=4 mice). Scale bar, 30 μm. Statistical significance (*P<0.05, **P<0.01) was assessed using two-sided unpaired t-test. Data are presented as mean ± SEM. The exact p-values and additional statistical details can be found in Supplementary table 12.

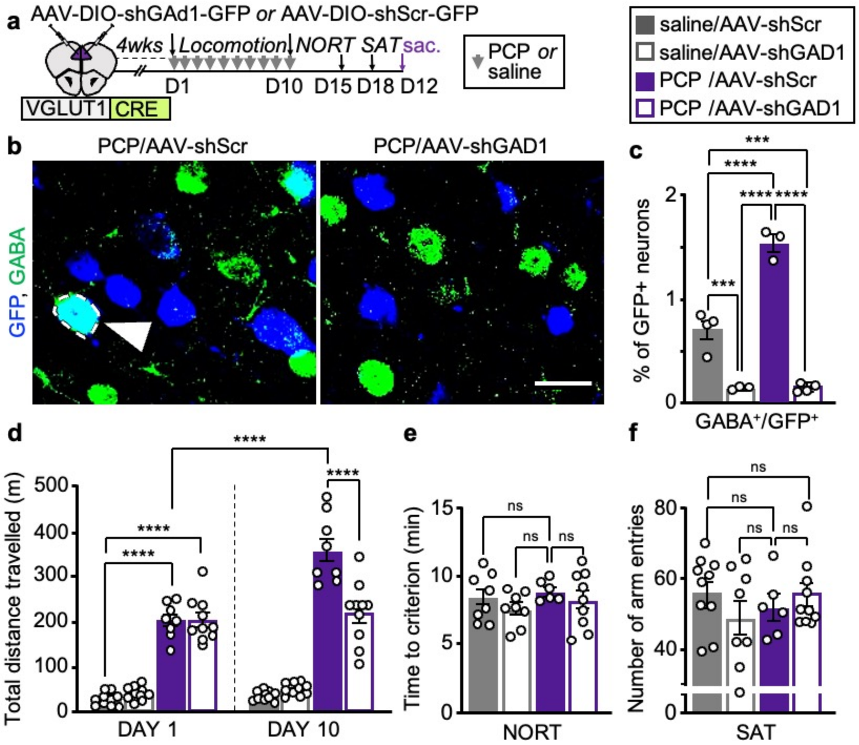

Supplementary Fig. 6 | Behavioral effects of shGAD1-mediated suppression of PCP-induced gain of GABA in PL glutamatergic neurons. a Experimental protocol to test whether overriding PCP-induced gain of GABA interferes with PCP-induced behavioral changes. **b** GABA is co-expressed in shScr^{+} but not shGAD1^{+} neurons (identified by GFP expression) in the PL. Scale bar, 20 µm. **c** shGAD1 suppresses GABA expression in glutamatergic neurons (*n*= from left to right 4, 3, 3, 4 mice). **d** PCP-induced locomotor sensitization is suppressed by shGAD1 (*n*= from left to right 10, 10, 8, 10 mice). **e**,**f** Time to criterion on the NORT and number of arm entries on the SAT are not altered by shGAD1 expression across experimental conditions (**e** *n*= from left to right 8, 8, 6, 9 mice; **f** *n*= from left to right 10, 8, 6, 10 mice). Statistical significance (***P<0.001, ****P<0.0001) was assessed using two-way ANOVA with Tukey's multiple-comparisons test (**c**-**e**), and ART factorial ANOVA followed by Mann Whitney test and Bonferroni correction (**f**). Data are presented as mean ± SEM. The exact p-values and additional statistical details can be found in Supplementary table 13.

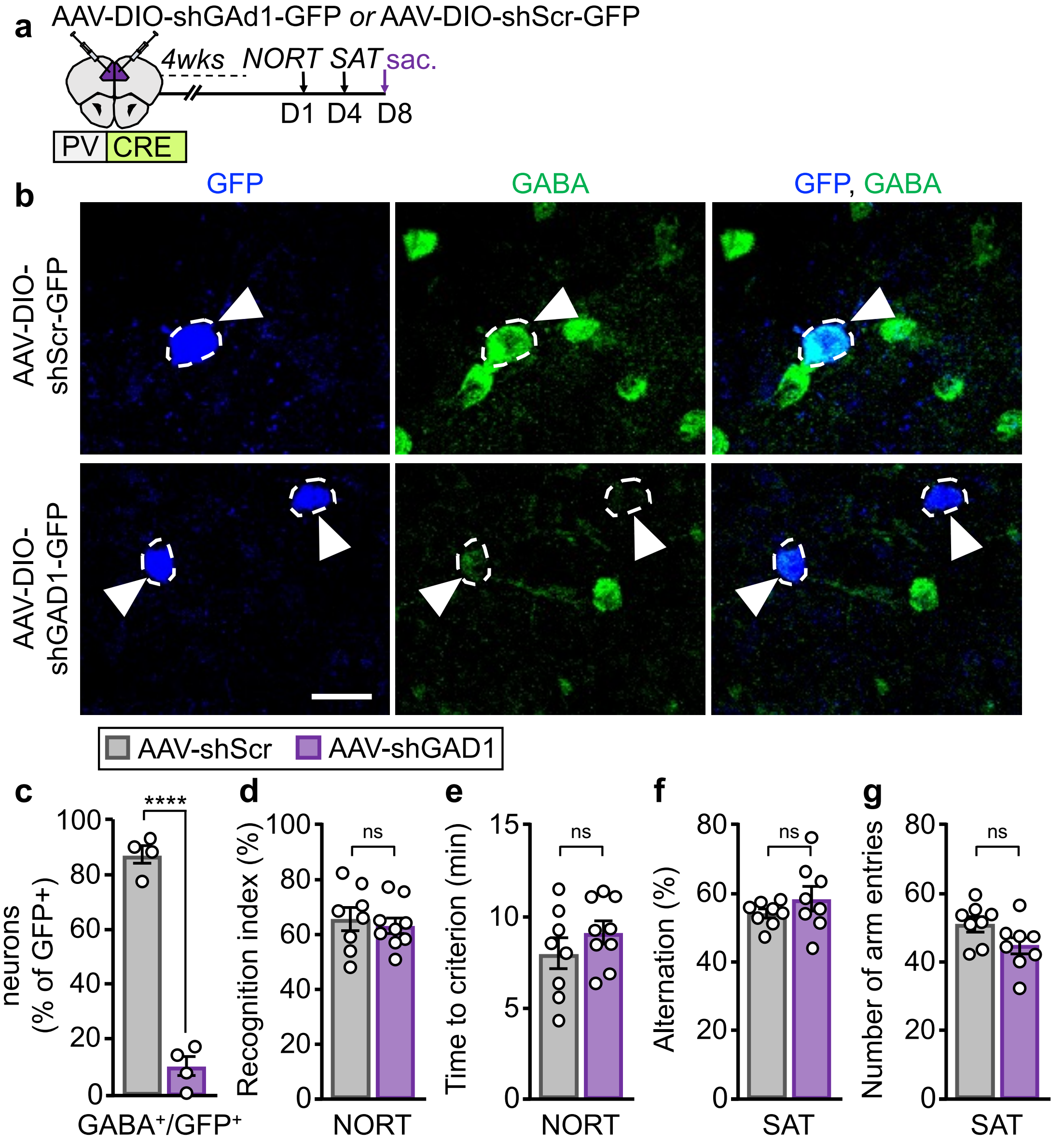

Supplementary Fig. 7 | Behavioral effects of shGAD1-mediated suppression of GABA in PL PV⁺ neurons in the absence of PCP-treatment. a Experimental protocol to test whether suppressing GABA expression in PL PV⁺ neurons contributes to the appearance of cognitive deficits in the absence of PCP-treatment. **b** GABA is co-expressed in shScr⁺ but not shGAD1⁺ PV⁺ neurons (identified by GFP expression) in the PL. Scale bar, 20 µm. **c** shGAD1 suppresses GABA expression in PV⁺ neurons (*n*=4 mice). **d-g** Recognition index and time to criterion on the NORT as well as alternation % and number of arm entries on the SAT are not altered by shGAD1-mediated suppression of GABA in PV⁺ PL neurons (**d,e** *n*= 8 AAV-shScr, 9 AAV-shGAD1 mice; **f,g** *n*= 8 mice). Statistical significance (****P<0.0001) was assessed using two-sided unpaired t-test. Data are presented as mean ± SEM. The exact p-values and additional statistical details can be found in Supplementary table 14.

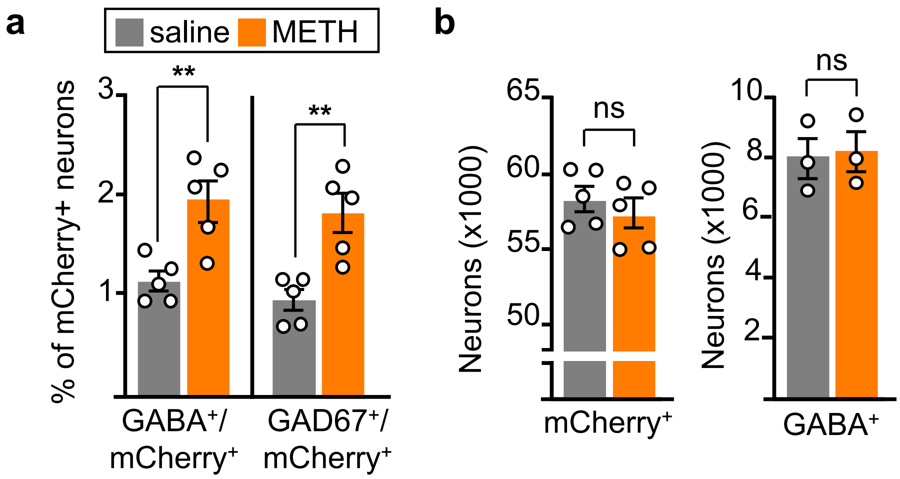

Supplementary Fig. 8| METH treatment increases the number of mCherry+ neurons co-expressing GABA and GAD67 in the PL, but not the total number of mCherry+ or GABA-only neurons. a Percent of GABA+/mCherry+ and GAD67+/mCherry+ cells in total mCherry+ neurons in the PL of METH-treated mice and saline controls (*n*=5 mice). **b** Quantification of mCherry+ and GABA+/mCherry- neurons in the PL of METH-treated mice and saline controls (*n*=5 mice (left graph); *n*=3 mice (right graph)). Statistical significance (**P<0.01) was assessed using two-sided unpaired t-test. Data are presented as mean ± SEM. The exact p-values and additional statistical details can be found in Supplementary table 15.

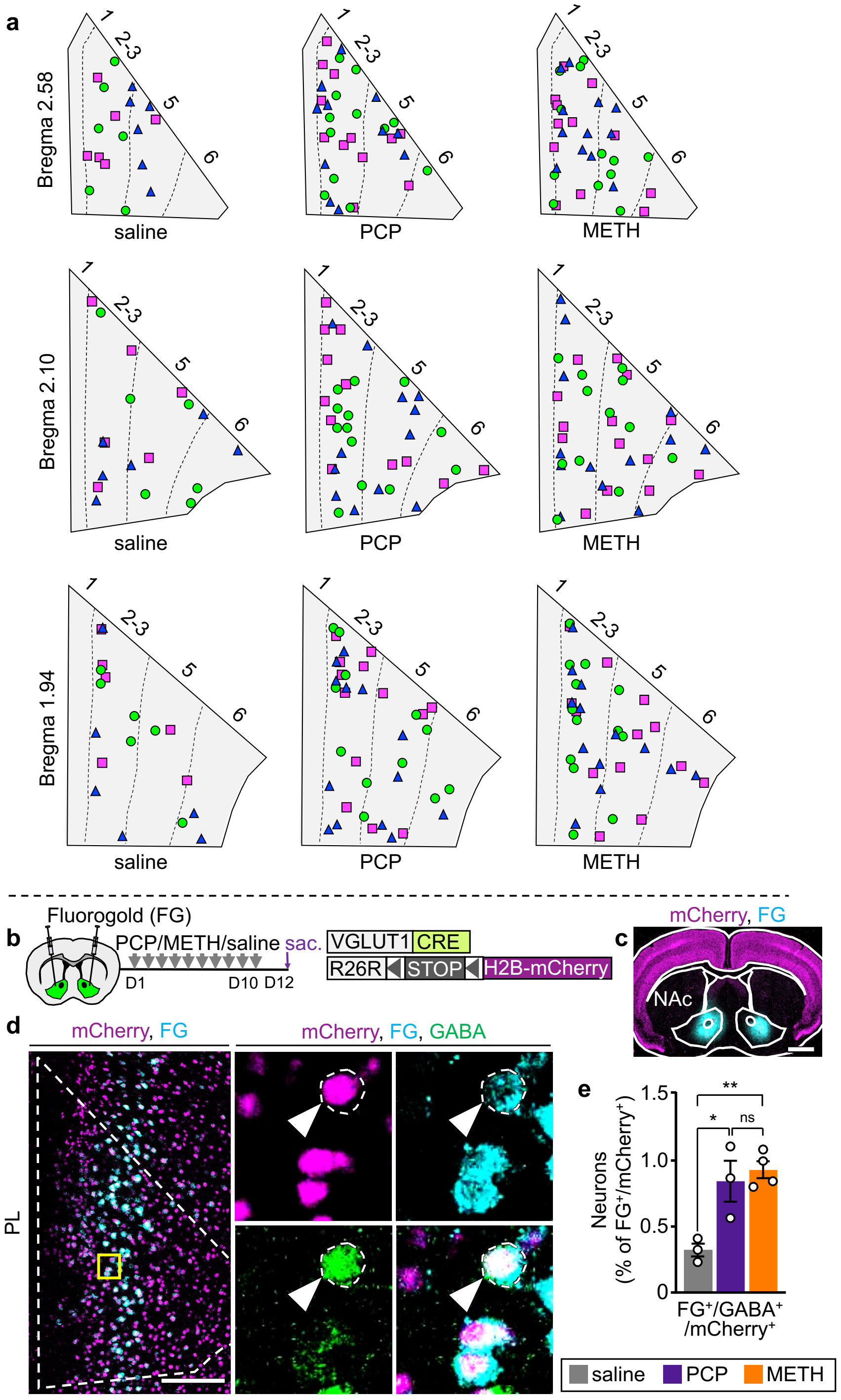

Supplementary Fig. 9| GAD1^{+}/VGLUT1^{+} neurons are enriched in layer 2/3 and layer 5 of the PL of PCP- and METH-treated mice and project to the nucleus accumbens. a Cartoons show the locations across PL layers and along the PL rostro-caudal axis of glutamatergic neurons that co-express GABA without drug treatment or switch transmitter identity upon treatment with PCP or METH. Each cartoon was obtained by superimposing the location of neurons from three distinct 30 μm PL sections, each belonging to a different mouse, and all positioned at the same distance from bregma. In each cartoon, dots of the same color and shape represent cells detected in the same mouse. Cartoons in the bottom row (Bregma 1.94) are also shown in Fig. **1d**, and Fig. **2d**. L1, layer 1; L2/3, layer 2/3; L5, layer 5; L6, layer 6. **b**

Experimental protocol to test whether PL neurons that gain GABA project to the NAc. **c** Fluorogold (FG) injection site in the NAc. Scale bar, 1 mm. **d** FG labeling in the PL. (Yellow rectangle) region illustrated at higher magnification on the right, showing a neuron co-expressing mCherry, GABA and FG. Scale bar, 200 μm. **e** Quantification of FG-expressing $GABA^+$/$mCherry^+$ neurons across treatments (*n*=3 saline, 3 PCP and 4 METH-treated mice). Statistical significance (*$P<0.05$, **$P<0.01$) was assessed using one-way ANOVA followed by Tukey's test. Data are presented as mean ± SEM. The exact p-values and additional statistical details can be found in Supplementary table 16.

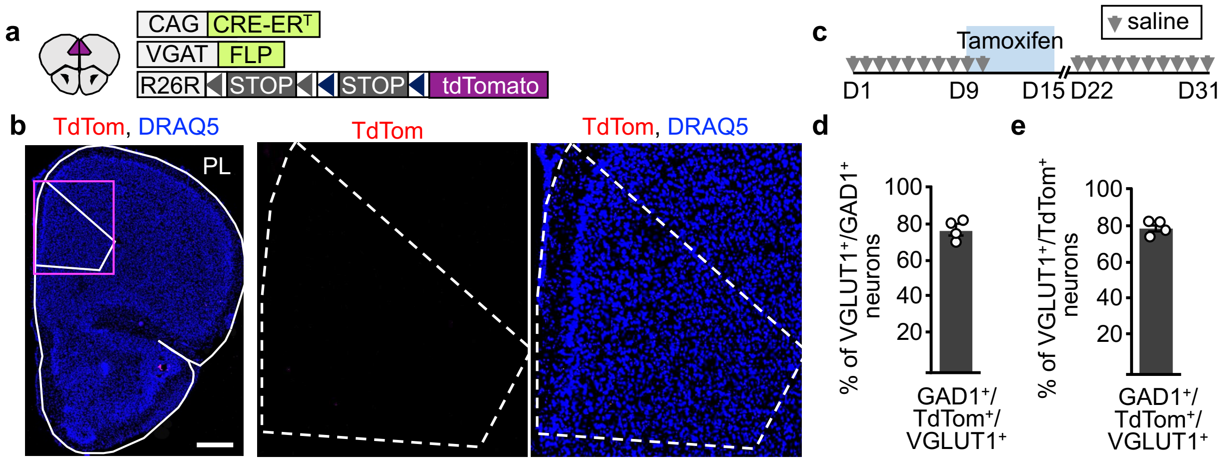

Supplementary Fig. 10 | The VGAT^{FLP}::CreER^{T}::TdTomato$^{cON/fON}$ mouse line allows labelling of neurons expressing VGAT at the time of tamoxifen administration. a Mouse line used for tamoxifen-inducible genetic labeling. **b** Representative images of the PL of a VGAT^{FLP}::CreER^{T}::TdTomato$^{cON/fON}$ mouse that did not receive tamoxifen treatment. In the absence of tamoxifen, no TdTomato labelling is present. (Magenta rectangle) regions shown at higher magnification on the right. Scale bar, 500 μm. **c** Experimental protocol to test efficiency and specificity of the mouse line. **d,e** Tamoxifen administration reveals the efficiency (77%, 1788 GAD1^{+}/VGLUT1^{+}/TdTom+ / 2328 GAD1^{+}/VGLUT1^{+} neurons) and specificity (79%, 1788 GAD1^{+}/VGLUT1^{+}/TdTom+ / 2268 VGLUT1+/TdTom+ neurons) of this approach in labelling GAD1^{+}/VGLUT1^{+} neurons with the TdTomato line (*n*=4 mice). Data are presented as mean ± SEM.

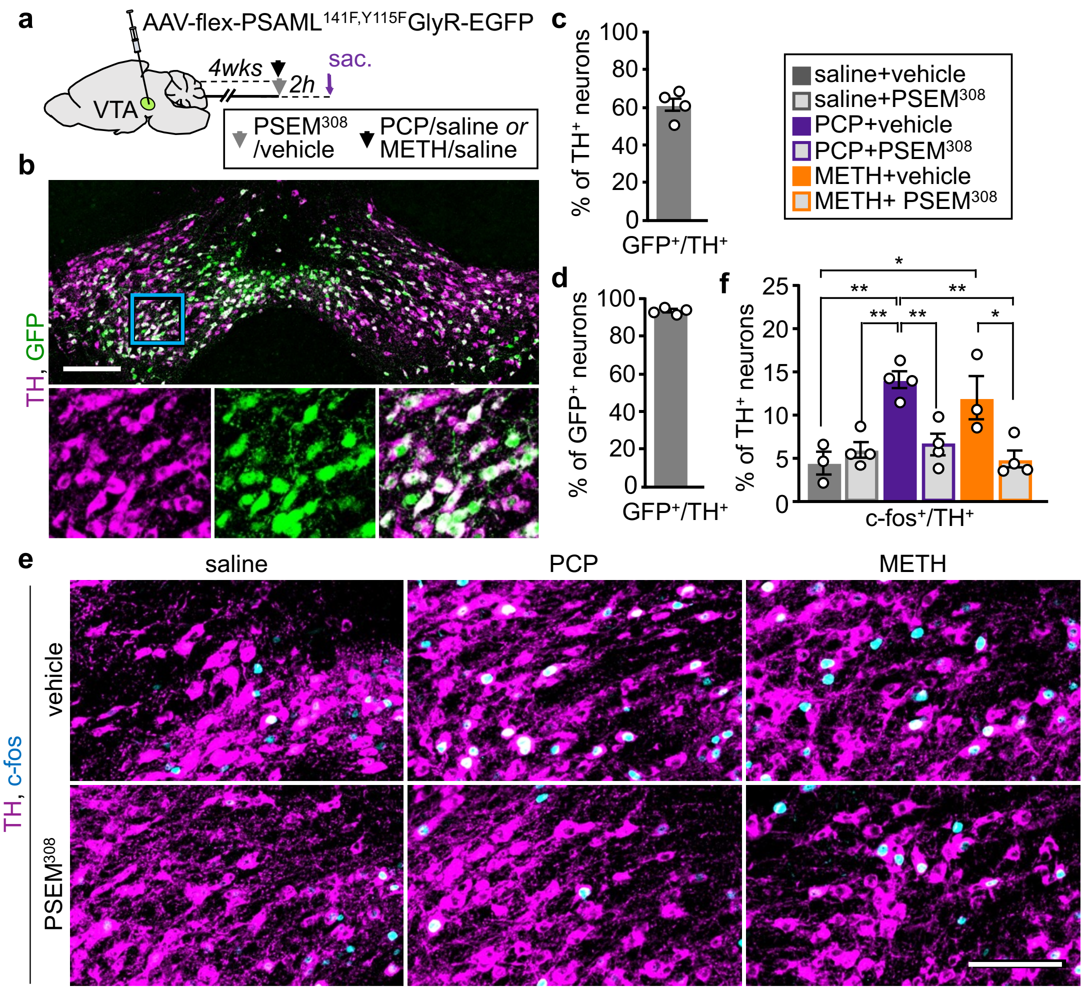

Supplementary Fig. 11 | Chemogenetic inhibition of VTA dopaminergic neurons suppresses their activity. a Experimental protocol to validate AAV-flex-PSAML-GlyR-GFP as a chemogenetic tool to inhibit VTA dopaminergic neurons. **b** Expression of TH and GFP in the VTA. (Blue rectangle) region shown at higher magnification below. Scale bar, 200 µm. **c** Efficiency of AAV transduction quantified as the percent of TH^+/GFP^+ neurons in the TH^+ population (62%, 3973/6455) (*n*=4 mice). **d** Specificity of AAV transduction quantified as the percent of TH^+/GFP^+ neurons in the GFP^+ population (92%, 3973/4297) (*n*=4 mice). **e** VTA expression of c-fos and TH across treatment groups. Scale bar, 200 µm. **f** Quantification of c-fos^+ expression in TH^+ neurons (*n*= from left to right 3, 4, 4, 4, 3, 4 mice). Statistical significance (*P<0.05, **P<0.01) was assessed using two-way ANOVA with Tukey's multiple-comparisons test. Data are presented as mean ± SEM. The exact p-values and additional statistical details can be found in Supplementary table 17.

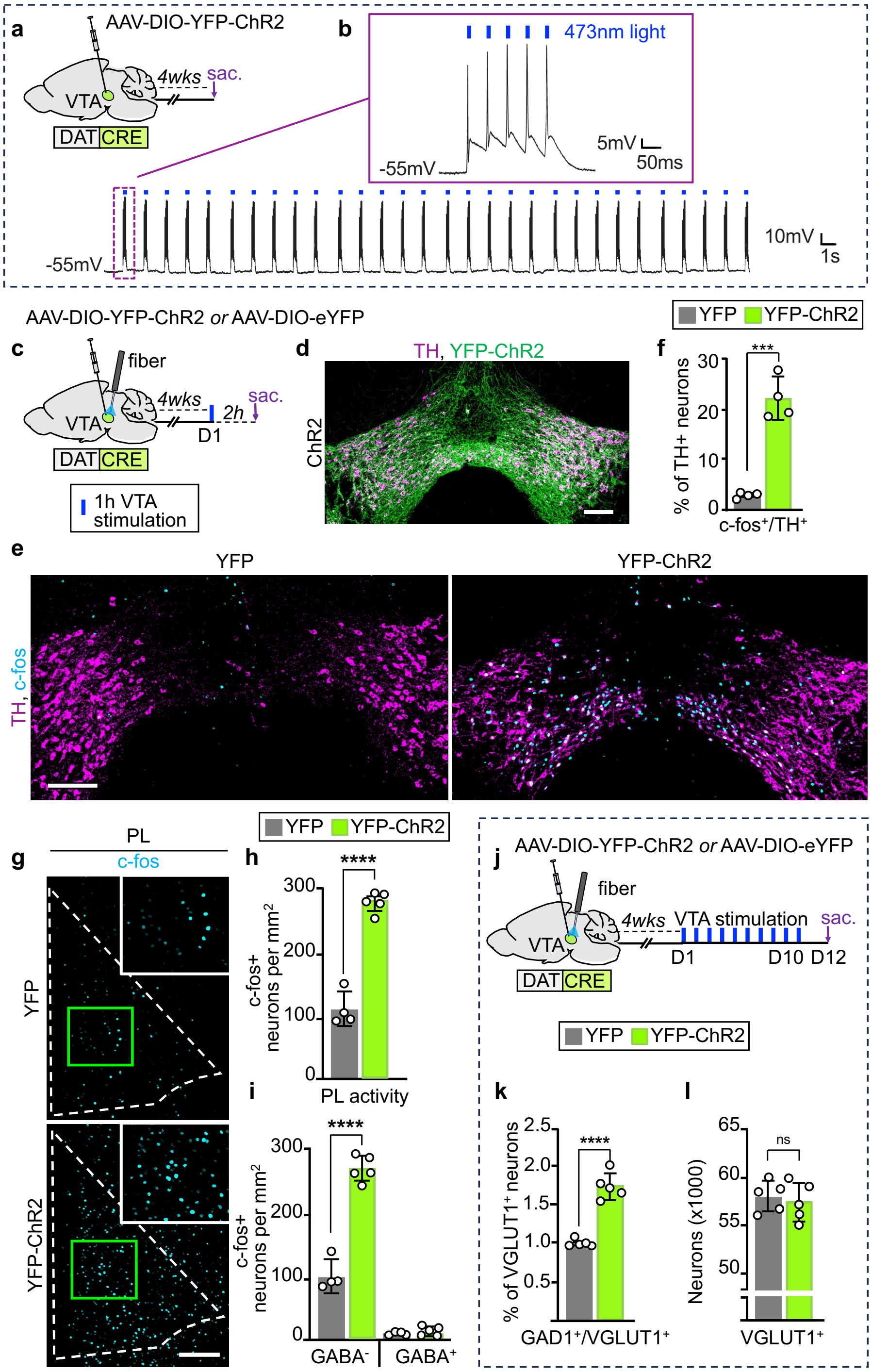

Supplementary Fig. 12 | Optogenetic stimulation activates VTA dopaminergic neurons and increases both c-fos expression in the PL and the percentage of PL GAD1^{+}/VGLUT1^{+} neurons without affecting the number of PL neurons expressing VGLUT1. a Experimental protocol for electrophysiological validation of ChR2 using optogenetic stimulation in acute brain slices. **b** Recording from ChR2^{+} neurons in acute VTA slices showing light-evoked phasic firing. (Purple rectangle) Traces illustrated at higher magnification. **c** Experimental protocol for *in vivo* optogenetic stimulation of VTA dopaminergic neurons. **d** Expression of YFP-ChR2 in the VTA. Scale bar, 200 μm. **e** c-fos and TH expression in the VTA following *in vivo* optogenetic stimulation. Scale bar, 200 μm. **f** Quantification of c-fos^{+}

expression in VTA TH^+ neurons 2h after the beginning of optogenetic stimulation (*n*=4 mice). **g** PL c-fos expression 2h after the beginning of *in vivo* optogenetic VTA stimulation. (Green rectangles) regions illustrated at higher magnification in insets. Scale bar, 250 μm. **h** Quantification of c-fos expression in (**g**) (*n*=4 YFP and 5 YFP-ChR2 mice). **i** c-fos labeling in $GABA^-$ and $GABA^+$ neurons (*n*=4 YFP and 5 YFP-ChR2 mice). **j** Experimental protocol to investigate the effect of repeated *in vivo* optogenetic VTA stimulation on PL neuron transmitter phenotype. **k** Percent of $GAD1^+$/$VGLUT1^+$ cells in total $VGLUT1^+$ neurons (*n*=5 mice). **l** Quantification of $VGLUT1^+$ neurons in the PL of ChR2 and YFP mice (*n*=5 mice). Statistical significance (***P<0.001, ****P<0.0001) was assessed using two-sided unpaired t-test (**f**,**h**,**k**,**l**), or two-way ANOVA with Tukey's multiple-comparisons test (**i**). Data are presented as mean ± SEM. The exact p-values and additional statistical details can be found in Supplementary table 18.

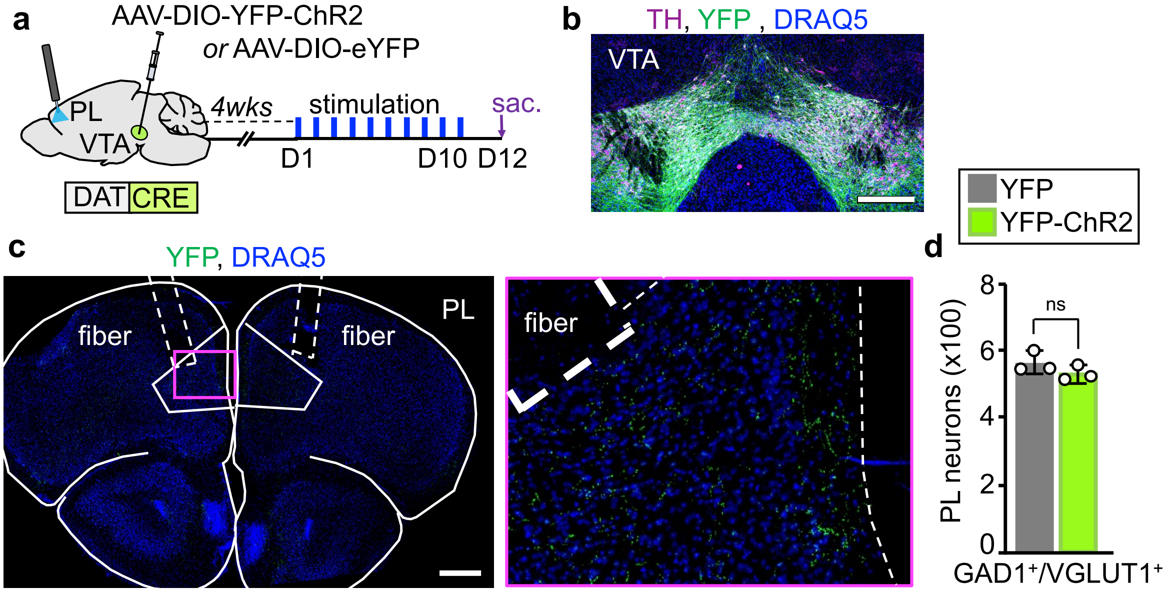

Supplementary Fig. 13 | Optogenetic stimulation of dopaminergic projections from the VTA to the PL does not affect the number of PL GAD1⁺/VGLUT1⁺ neurons. a Experimental protocol for optogenetic stimulation of dopaminergic projections from the VTA to the PL. **b** Expression of YFP-ChR2 in the VTA. Scale bar, 200 µm. **c** Representative images of ChR2⁺-YFP fibers in the PL and position of optic fibers above the PL. The magenta rectangle indicates regions shown at higher magnification on the right. Scale bar, 500 µm. **d** Quantification of GAD1⁺/VGLUT1⁺ neurons in the PL after optogenetic stimulation of VTA-to-PL dopaminergic projections (*n*=3 mice). Statistical significance was assessed using two-sided unpaired t-test. Data are presented as mean ± SEM. Additional statistical details can be found in Supplementary table 19.

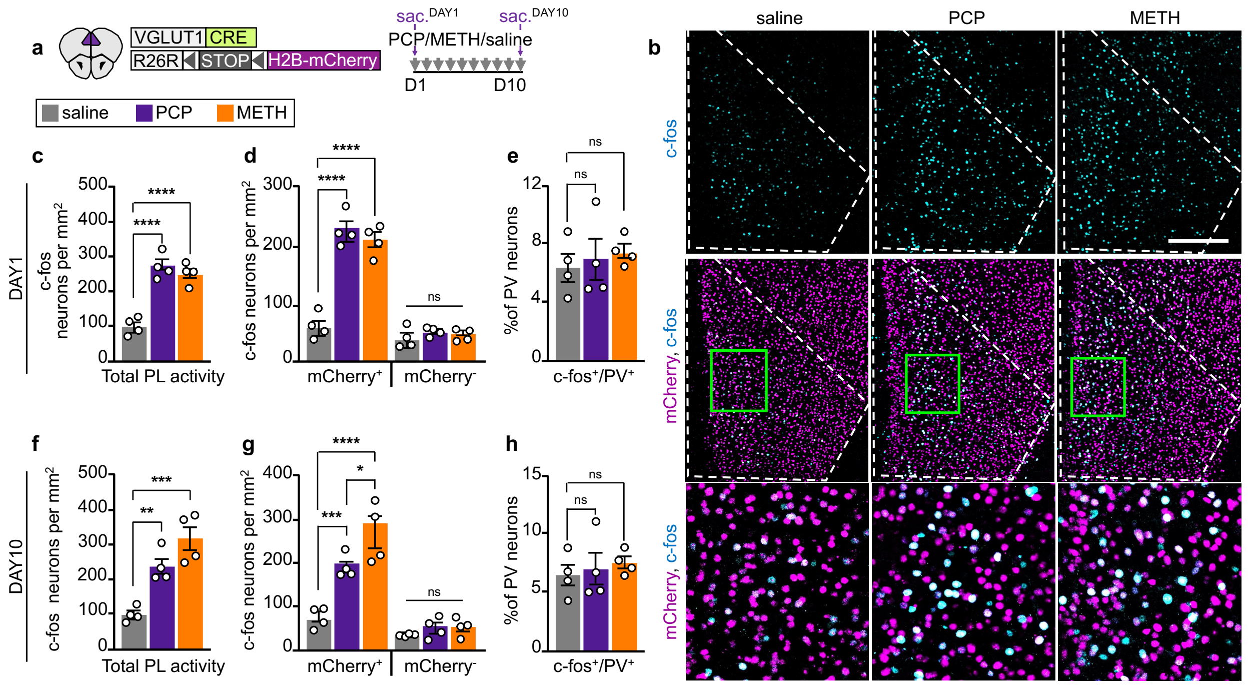

Supplementary Fig. 14 | Both PCP and METH increase neuronal activity in PL glutamatergic neurons. a Experimental protocol to determine the effect of drug treatment on c-fos expression in PL glutamatergic neurons. **b** PL c-fos-labeled mCherry+ cells 2h after single drug injection on day 1. (Green rectangles) regions illustrated at higher magnification below. Scale bar, 250 μm. **c**,**f** Quantification of c-fos+ PL neurons on day 1 (**c**) (*n*=4 mice) and day 10 (**f**) (*n*=4mice). **d**,**g** Quantification of c-fos labeling in mCherry+ and mCherry- neurons at the same time points (*n*=4 mice). **e**,**h** c-fos labeling of PV+ neurons at the same time points (*n*=4 mice). Statistical significance (*P<0.05, **P<0.01, ***P<0.001, ****P<0.0001) was assessed using one-way ANOVA with Dunnett's multiple-comparisons test (**c**,**e**,**f**,**h**) or two-way ANOVA with Tukey's multiple-comparisons test (**d**,**g**). Data are presented as mean ± SEM. The exact p-values and additional statistical details can be found in Supplementary table 20.

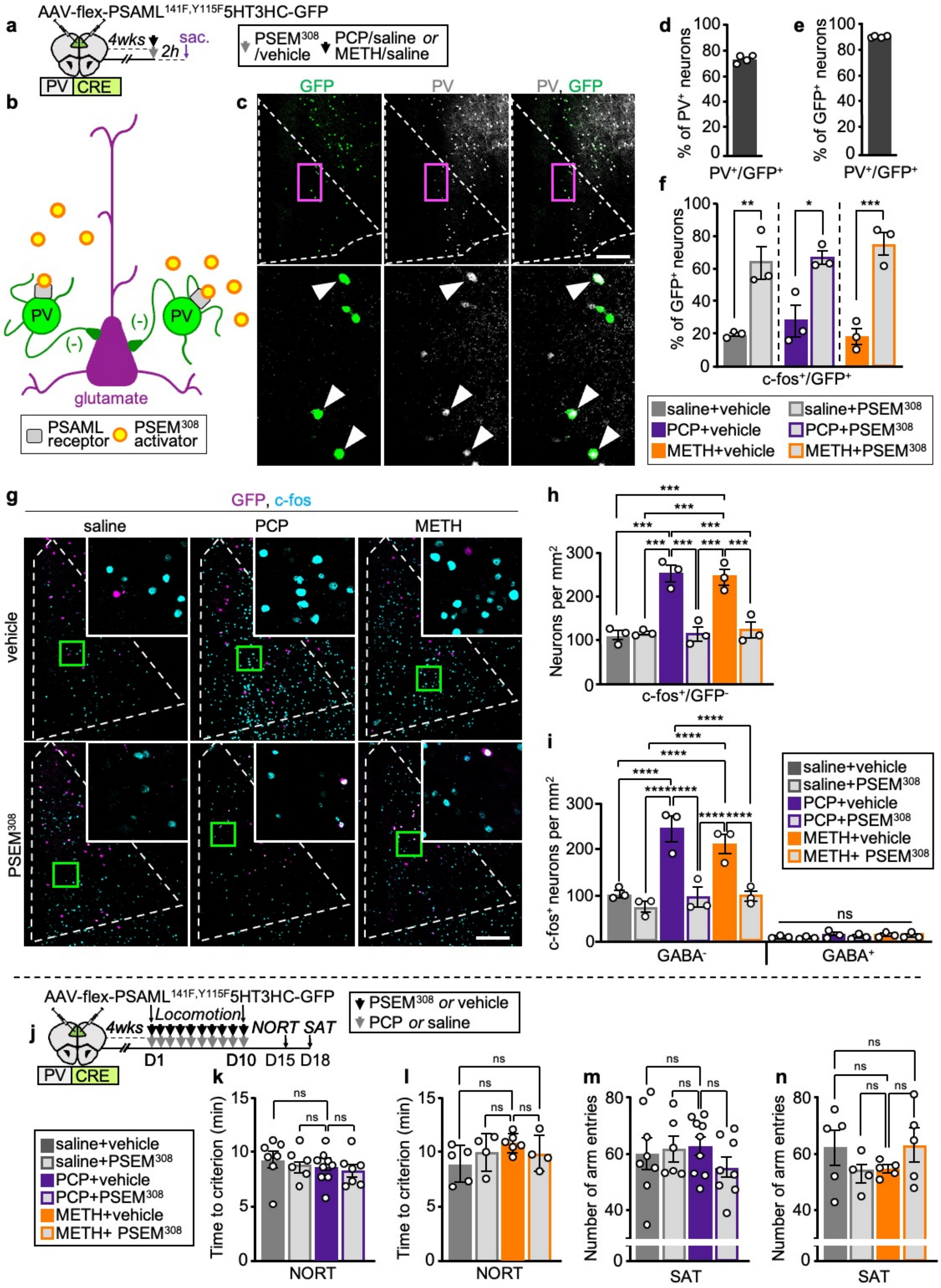

Supplementary Fig. 15 | Chemogenetic stimulation of PV⁺ interneurons suppresses drug-induced PL hyperactivity and does not affect exploratory behaviors. a Experimental protocol to validate AAV-flex-PSAML-5HT3HC-GFP as a chemogenetic tool to activate PL PV⁺ neurons and suppress drug-induced

PL hyperactivity. **b** Strategy to suppress drug-induced hyperactivity of PL glutamatergic neurons. **c** Expression of PSAML-5HT3HC-GFP in PL PV^+ neurons in a mouse sacrificed 2h after acute injection of METH and vehicle. (Magenta rectangles) regions shown at higher magnification below. Arrowheads indicate PV^+ neurons expressing GFP. Scale bar, 200 μm. **d** Efficiency of AAV transduction measured as the percent of PV^+/GFP^+ neurons in the PV^+ population (74%, 906/1229) (*n*=4 mice). **e** Specificity of AAV transduction measured as the percent of PV^+/GFP^+ neurons in the GFP^+ population (91%, 906/998) (*n*=4 mice). **f** Quantification of c-fos expression in AAV-transduced neurons (identified by GFP) 2h after injections (*n*=3 mice). **g** PL neurons expressing c-fos, 2h after drug injections. (Green rectangles) regions illustrated at higher magnification in insets. Scale bar, 250 μm. **h** Quantification of c-fos expression in PL neurons not expressing GFP (*n*=3 mice). **i** Quantification of c-$fos^+/GABA^+$ and c-$fos^+/GABA^-$ (ostensibly glutamatergic) PL neurons 2h after injections (*n*=3 mice). **j** Experimental protocol to assess the effect of chemogenetic activation of PV^+ neurons on exploratory behaviors. **k-n** Time to criterion on the NORT and number of arm entries on the SAT are not altered by chemogenetic activation of PV^+ neurons across experimental conditions (**k** *n*= from left to right 7, 6, 9, 7 mice; **l** *n*= from left to right 5, 4, 6, 4 mice; **m** *n*= from left to right 8, 6, 9, 8 mice; **n** *n*= from left to right 5, 4, 5, 5 mice). Statistical significance (*P<0.05, **P<0.01, ***P<0.001, ****P<0.0001) was assessed using two-way ANOVA with Tukey's multiple-comparisons test. Data are presented as mean ± SEM. The exact p-values and additional statistical details can be found in Supplementary table 21.

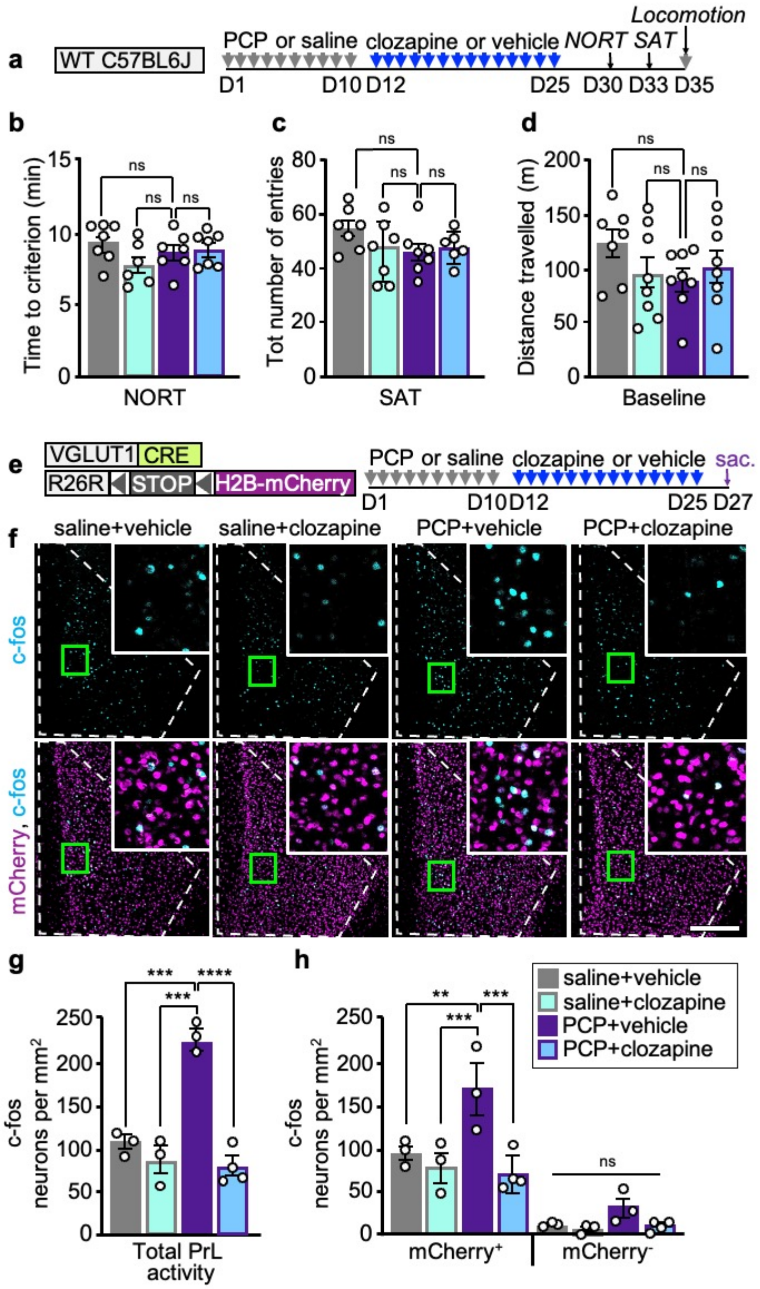

Supplementary Fig. 16 | Clozapine treatment does not affect exploratory behaviors and normalizes PL hyperactivity after PCP-treatment. a Experimental protocol to test the of consecutive treatment with PCP and clozapine on exploratory behaviors. **b**-**d** Time to criterion on the NORT, number of arm entries on the SAT and baseline locomotion are unchanged across treatment groups (**b** *n*= from left to right 7, 6, 7, 7 mice; **c** *n*= from left to right 7, 7, 7, 6 mice; **d** *n*= from left to right 7, 8, 8, 8 mice). **e** Experimental protocol to determine if consecutive treatment with PCP and clozapine affects c-fos expression in the PL. **f** PL c-fos$^+$ neurons across treatments. (Green rectangles) regions illustrated at higher magnification in insets. Scale bar, 250 μm. **g**,**h** Clozapine normalizes PL c-fos$^+$ expression after the end PCP treatment (*n*= from left to right 3, 3, 3, 4 mice). Statistical significance (**P<0.01, ***P<0.001, ****P<0.0001) was assessed by two-way ANOVA with Tukey's multiple-comparisons test. Data are presented as mean ± SEM. The exact p-values and additional statistical details can be found in Supplementary table 22.

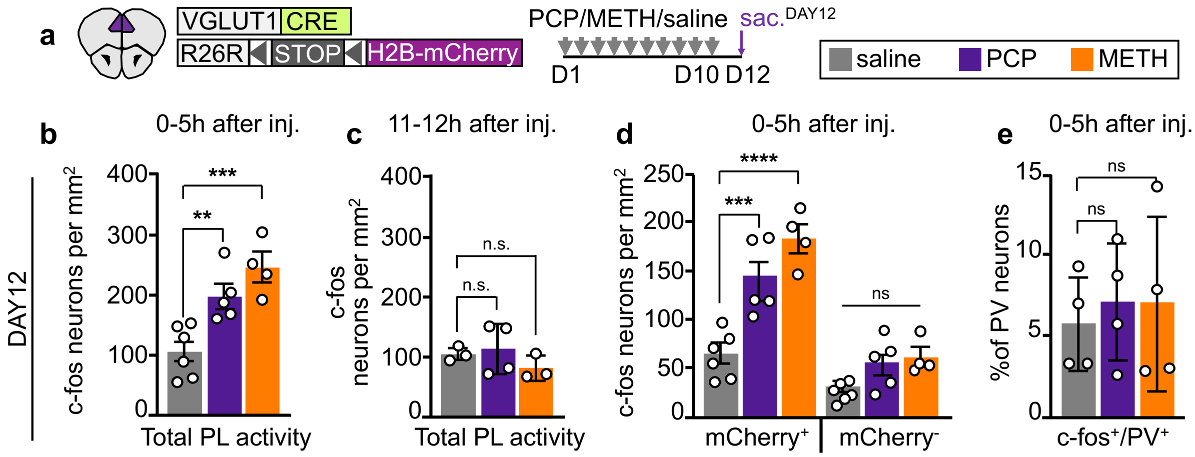

Supplementary Fig. 17 | c-fos expression in PL glutamatergic neurons is increased in a time-of-day dependent manner after 2 days of drug-washout. a Experimental protocol to determine the effect of drug washout on c-fos expression in PL glutamatergic neurons. **b** Quantification of c-fos$^+$ PL neurons 2 days after the end of treatment, in mice sacrificed 0-to-5 hours after the time of day when they previously received the drugs (*n*= from left to right 6, 5, 4 mice). **c** Quantification of c-fos$^+$ PL neurons 2 days after the end of treatment, in mice sacrificed 11-to-12 hours after the time of day when they previously received the drugs (*n*= from left to right 3, 4, 3 mice). **d** Quantification of c-fos labeling in mCherry$^+$ and mCherry$^-$ neurons at the same time point of (**b**) (*n*= from left to right 6, 5, 4 mice). **e** c-fos labeling of PV$^+$ neurons at the same time point of (**b**) (*n*=4 mice). Statistical significance (**P<0.01, ***P<0.001, ****P<0.0001) was assessed using one-way ANOVA with Dunnett's multiple-comparisons test (**b**,**c**,**e**) or two-way ANOVA with Tukey's multiple-comparisons test (**d**). Data are presented as mean ± SEM. The exact p-values and additional statistical details can be found in Supplementary table 23.

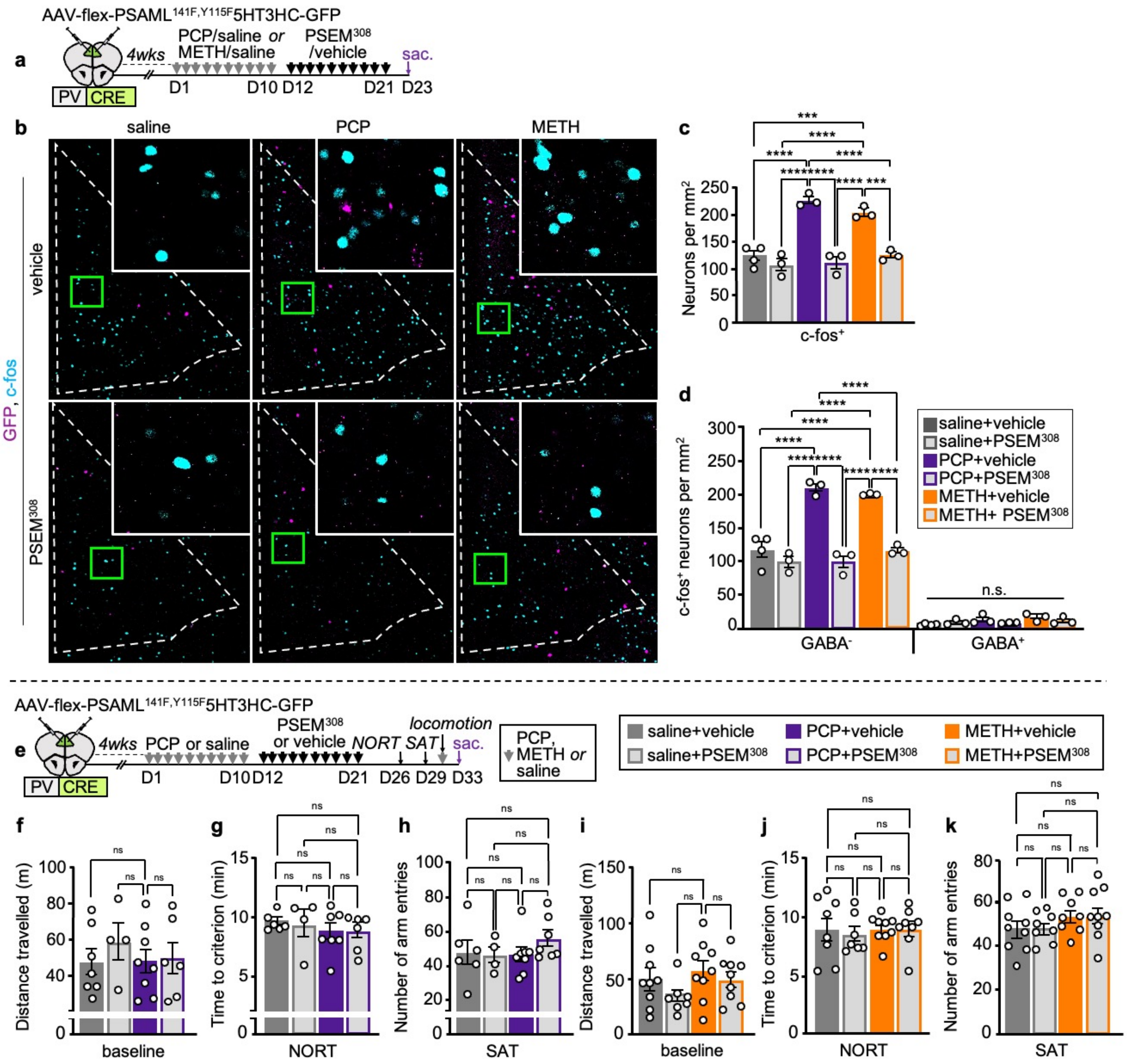

Supplementary Fig. 18 | Chemogenetic activation of PL PV⁺ neurons after the end of PCP- or METH-treatment normalizes PL activity, and does not affect exploratory behaviors. a Experimental protocol to manipulate PL activity after the end of drug-treatment. **b** c-fos⁺ and GFP⁺ PL neurons across treatments. (Green rectangles) regions illustrated at higher magnification in insets. Scale bar, 250 μm. **c** c-fos expression in the PL of mice that received drug-treatment followed by 10 daily injections with PSEM^{308} or vehicle as control (*n*= from left to right 4, 3, 3, 3, 3, 3 mice). **d** c-fos⁺ neurons in GABA⁺ and GABA⁻ neurons (*n*= from left to right 4, 3, 3, 3, 3, 3 mice). **e** Experimental protocol to test the behavioral effect of repeatedly activating PL PV⁺ neurons after the end of PCP-treatment. **f**-**k** No changes in baseline locomotion, time to criterion on NORT or number of arm entries on SAT were observed across conditions (**f** *n*= from left to right 7, 4, 8, 6 mice; **g** *n*= from left to right 6, 4, 7, 6 mice; **h** *n*= from left to right 6, 4, 8, 7 mice; **f** *n*= from left to right 7, 4, 8, 6 mice; **i** *n*= from left to right 9, 7, 9, 9 mice; **j** *n*= from left to right 8, 7, 9, 8 mice; **k** *n*= from left to right 9, 8, 8, 9 mice). Statistical significance (***P<0.001, ****P<0.0001) was assessed using two-way ANOVA with Tukey's multiple-comparisons test. Data are presented as mean ± SEM. The exact p-values and additional statistical details can be found in Supplementary table 24.

Supplementary table 1. Statistical details of Fig. 1

Figure	Data/Comparison	N, n/group	Primary statistic	Post-hoc test	Comparison	p value	Notation	F/t statistic
1c (left)	Mean number of mCherry+/GABA+ neurons. Between treatment groups.	N=10 mice, 5/group	Unpaired t-test		saline vs. PCP	<0.0001	****	t=7.722, df=8
1c (right)	Mean number of mCherry+/GAD67+ neurons. Between treatment groups.	N=10 mice, 5/group	Unpaired t-test		saline vs. PCP	0.0007	***	t=5.370, df=8
1g	Percentage of cell area occupied by VGAT RNA puncta (3 mice, 25 cells per cell type/mouse). Between groups of different neuronal types in PCP-treated mice.	N=3 mice, 3/group	Kruskal-Wallis test		Mean effect of group	<0.0001	****	Kruskal-Wallis statistic: 148.7
				Dunn's	mCherry+ only vs. mCherry+/GAD1+	<0.0001	****	
				Dunn's	mCherry+ only vs. GAD1+ only	<0.0001	****	
				Dunn's	mCherry+/GAD1+ vs. GAD1+ only	>0.9999	ns	
1h	Percentage of cell area occupied by VGLUT1 RNA puncta (3 mice, 25 cells per cell type/mouse). Between groups of different neuronal types in PCP-treated mice.	N=3 mice, 3/group	Kruskal-Wallis test		Mean effect of group	<0.0001	****	Kruskal-Wallis statistic: 152.7
				Dunn's	mCherry+ only vs. mCherry+/GAD1+	<0.0001	****	
				Dunn's	mCherry+ only vs. GAD1+ only	<0.0001	****	
				Dunn's	mCherry+/GAD1+ vs. GAD1+ only	<0.0001	****	
1k	Mean number of PL VGLUT1+/GAD1+ neurons. Between treatment groups.	N=16 mice, 4/group	two-way ANOVA		Interaction	<0.0001	****	F (1, 12) = 80.97
					Row Factor (effect of drug treatment)	<0.0001	****	F (1, 12) = 82.50
					Column Factor (effect of AAV)	<0.0001	****	F (1, 12) = 256.3
				Tukey's	saline/AAV-shScr vs. saline/AAV-shGAD1	0.0016	**	
				Tukey's	saline/AAV-shScr vs. PCP/AAV-shScr	<0.0001	****	
				Tukey's	saline/AAV-shScr vs. PCP/AAV-shGAD1	0.0018	**	
				Tukey's	saline/AAV-shGAD1 vs. PCP/AAV-shScr	<0.0001	****	
				Tukey's	saline/AAV-shGAD1 vs. PCP/AAV-shGAD1	>0.9999	ns	
				Tukey's	PCP/AAV-shScr vs. PCP/AAV-shGAD1	<0.0001	****	
1l	(Total distance travelled after the last of 10 PCP injections administered once a day for 10 days) - (total distance travelled after the first PCP injection). Between treatment & time groups.	N=38 mice, 8-10/group	two-way ANOVA		Interaction	0.0001	***	F (1, 34) = 19.09
					Row Factor (effect of drug treatment)	<0.0001	****	F (1, 34) = 23.55
					Column Factor (effect of AAV)	<0.0001	****	F (1, 34) = 19.85
				Tukey's	saline/AAV-shScr vs. saline/AAV-shGAD1	>0.9999	ns	
				Tukey's	saline/AAV-shScr vs. PCP/AAV-shScr	<0.0001	****	
				Tukey's	saline/AAV-shScr vs. PCP/AAV-shGAD1	0.9914	ns	
				Tukey's	saline/AAV-shGAD1 vs. PCP/AAV-shScr	<0.0001	****	
				Tukey's	saline/AAV-shGAD1 vs. PCP/AAV-shGAD1	0.9848	ns	
				Tukey's	PCP/AAV-shScr vs. PCP/AAV-shGAD1	<0.0001	****	
1m	Mean recognition index % in the NORT. Between treatment groups.	N=31mice, 6-9/group	two-way ANOVA		Interaction	<0.0001	****	F (1, 27) = 25.19
					Row Factor (effect of drug treatment)	0.001	**	F (1, 27) = 13.53
					Column Factor (effect of AAV)	0.0054	**	F (1, 27) = 9.157
				Tukey's	saline/AAV-shScr vs. saline/AAV-shGAD1	0.4817	ns	
				Tukey's	saline/AAV-shScr vs. PCP/AAV-shScr	<0.0001	****	
				Tukey's	saline/AAV-shScr vs. PCP/AAV-shGAD1	0.9613	ns	
				Tukey's	saline/AAV-shGAD1 vs. PCP/AAV-shScr	0.0006	***	
				Tukey's	saline/AAV-shGAD1 vs. PCP/AAV-shGAD1	0.7491	ns	
				Tukey's	PCP/AAV-shScr vs. PCP/AAV-shGAD1	<0.0001	****	
1n	Mean alternation % in the SAT. Between treatment groups.	N=34 mice, 6-10/group	two-way ANOVA		Interaction	0.0851	ns	F (1, 30) = 3.170
					Row Factor (effect of drug treatment)	0.0005	***	F (1, 30) = 14.96
					Column Factor (effect of AAV)	0.003	**	F (1, 30) = 10.45
				Tukey's	saline/AAV-shScr vs. saline/AAV-shGAD1	0.7078	ns	
				Tukey's	saline/AAV-shScr vs. PCP/AAV-shScr	0.0032	**	
				Tukey's	saline/AAV-shScr vs. PCP/AAV-shGAD1	0.959	ns	
				Tukey's	saline/AAV-shGAD1 vs. PCP/AAV-shScr	0.0004	***	
				Tukey's	saline/AAV-shGAD1 vs. PCP/AAV-shGAD1	0.4255	ns	
				Tukey's	PCP/AAV-shScr vs. PCP/AAV-shGAD1	0.0097	**	
1o	Correlation between number of VGLUT1+/GAD1+ neurons and locomotor sesitizatiuon measured as distance travelled on D10-D1.	N=16 mice, 4/treatment	Linear regression and Pearson's correlation analysis			0.0002	***	R squared= 0.6485
1p	Correlation between number of VGLUT1+/GAD1+ neurons and recongition index in the	N=16 mice, 4/treatment	Linear regression and Pearson's correlation analysis			0.0019	**	R squared= 0.5107
1q	Correlation between number of VGLUT1+/GAD1+ neurons and alternation in the SAT.	N=16 mice, 4/treatment	Linear regression and Pearson's correlation analysis			<0.0001	****	R squared= 0.6856

Supplementary table 2. Statistical details of Fig. 2

Figure	Data/Comparison	N, n/group	Primary statistic	Post-hoc test	Comparison	p value	Notation	F/t statistic
**2c (left)**	Mean number of mCherry+/GABA+ neurons. Between treatment groups.	N=10 mice, 5/group	Unpaired t-test		saline vs. METH	0.0044	**	t=3.918, df=8
**2c (right)**	Mean number of mCherry+/GAD67+ neurons. Between treatment groups.	N=10 mice, 5/group	Mann Whitney		saline vs. METH	0.0079	**	
**2e**	Percentage of cell area occupied by VGAT RNA puncta (3 mice, 25 cells per cell type/mouse). Between groups of different neuronal types in METH-treated mice.	N=3 mice, 3/group	Kruskal-Wallis test		Mean effect of group	<0.0001	****	Kruskal-Wallis statistic: 150.6
				Dunn's	mCherry+ only vs. mCherry+/GAD1+	<0.0001	****	
				Dunn's	mCherry+ only vs. GAD1+ only	<0.0001	****	
				Dunn's	mCherry+/GAD1+ vs. GAD1+ only	>0.9999	ns	
**2f**	Percentage of cell area occupied by VGLUT1 RNA puncta (3 mice, 25 cells per cell type/mouse). Between groups of different neuronal types in METH-treated mice.	N=3 mice, 3/group	Kruskal-Wallis test		Mean effect of group	<0.0001	****	Kruskal-Wallis statistic: 156.2
				Dunn's	mCherry+ only vs. mCherry+/GAD1+	<0.0001	****	
				Dunn's	mCherry+ only vs. GAD1+ only	<0.0001	****	
				Dunn's	mCherry+/GAD1+ vs. GAD1+ only	<0.0001	****	

Supplementary table 3. Statistical details of Fig. 3.

Figure	Data/Comparison	N, n/group	Primary statistic	Post-hoc test	Comparison	p value	Notation	F/t statistic
**3d**	Mean number of VGLUT1+/GAD1+/TdTom+, VGLUT1+/GAD1+ and VGLUT1+/TdTom+ neurons. Between treatment groups.	N=8 mice, 4/group	two-way ANOVA		Interaction	<0.0001	****	F (2, 18) = 47.70
					Row Factor (cell type)	<0.0001	****	F (2, 18) = 54.06
					Column Factor (effect of treatment)	<0.0001	****	F (1, 18) = 41.71
				Tukey's	VGLUT1+/GAD+/TdTomato (saline+saline) vs. VGLUT1+/GAD+/TdTomato (saline+PCP)	>0.9999	ns	
				Tukey's	VGLUT1+/GAD+/TdTomato (saline+saline) vs. VGLUT1+/GAD1+ (saline+saline)	0.0002	***	
				Tukey's	VGLUT1+/GAD+/TdTomato (saline+saline) vs. VGLUT1+/GAD1+ (saline+PCP)	0.0002	***	
				Tukey's	VGLUT1+/GAD+/TdTomato (saline+saline) vs. VGLUT1+/TdTomato (saline+saline)	<0.0001	****	
				Tukey's	VGLUT1+/GAD+/TdTomato (saline+saline) vs. VGLUT1+/TdTomato (saline+PCP)	<0.0001	****	
				Tukey's	VGLUT1+/GAD+/TdTomato (saline+PCP) vs. VGLUT1+/GAD1+ (saline+saline)	0.0002	***	
				Tukey's	VGLUT1+/GAD+/TdTomato (saline+PCP) vs. VGLUT1+/GAD1+ (saline+PCP)	0.0002	***	
				Tukey's	VGLUT1+/GAD+/TdTomato (saline+PCP) vs. VGLUT1+/TdTomato (saline+saline)	0.0001	***	
				Tukey's	VGLUT1+/GAD+/TdTomato (saline+PCP) vs. VGLUT1+/TdTomato (saline+PCP)	<0.0001	****	
				Tukey's	VGLUT1+/GAD1+ (saline+saline) vs. VGLUT1+/GAD1+(saline+PCP)	<0.0001	****	
				Tukey's	VGLUT1+/GAD1+ (saline+saline) vs. VGLUT1+/TdTomato (saline+saline)	0.9997	ns	
				Tukey's	VGLUT1+/GAD1+ (saline+saline) vs. VGLUT1+/TdTomato (saline+PCP)	0.981	ns	
				Tukey's	VGLUT1+/GAD1+ (saline+PCP) vs. VGLUT1+/TdTomato (saline+saline)	<0.0001	****	
				Tukey's	VGLUT1+/GAD1+ (saline+PCP) vs. VGLUT1+/TdTomato (saline+PCP)	<0.0001	****	
				Tukey's	VGLUT1+/TdTomato (saline+saline) vs. VGLUT1+/TdTomato (saline+PCP)	0.9984	ns	
**3f**	Mean number of VGLUT1+/GAD1+/TdTom+,VGLUT1+/GAD1+ and VGLUT1+/TdTom+ neurons. Between treatment groups.	N=13 mice, 4-5/group	two-way ANOVA		Interaction	0.3332	ns	F (4, 30) = 1.195
					Row Factor (cell type)	<0.0001	****	F (2, 30) = 96.99
					Column Factor (effect of treatment)	0.9048	ns	F (2, 30) = 0.1004
				Tukey's	GAD1+/TdTom+/VGLUT1+ (PCP+saline) vs. GAD1+/TdTom+/VGLUT1+ (PCP+METH)	ns	>0.9999	
				Tukey's	GAD1+/TdTom+/VGLUT1+ (PCP+saline) vs.	ns	>0.9999	
				Tukey's	GAD1+/TdTom+/VGLUT1+ (PCP+saline) vs. GAD1+/VGLUT1+ (PCP+saline)	****	<0.0001	
				Tukey's	GAD1+/TdTom+/VGLUT1+ (PCP+saline) vs. GAD1+/VGLUT1+ (PCP+METH)	****	<0.0001	
				Tukey's	GAD1+/TdTom+/VGLUT1+ (PCP+saline) vs. GAD1+/VGLUT1+ (PCP+PCP)	**	0.0014	
				Tukey's	GAD1+/TdTom+/VGLUT1+ (PCP+saline) vs. TdTom+/VGLUT1+ (PCP+saline)	****	<0.0001	
				Tukey's	GAD1+/TdTom+/VGLUT1+ (PCP+saline) vs. TdTom+/VGLUT1+ (PCP+METH)	****	<0.0001	
				Tukey's	GAD1+/TdTom+/VGLUT1+ (PCP+saline) vs. TdTom+/VGLUT1+ (PCP+PCP)	****	<0.0001	
				Tukey's	GAD1+/TdTom+/VGLUT1+ (PCP+METH) vs. GAD1+/TdTom+/VGLUT1+	ns	>0.9999	
				Tukey's	GAD1+/TdTom+/VGLUT1+ (PCP+METH) vs. GAD1+/VGLUT1+ (PCP+saline)	****	<0.0001	
				Tukey's	GAD1+/TdTom+/VGLUT1+ (PCP+METH) vs. GAD1+/VGLUT1+ (PCP+METH)	****	<0.0001	
				Tukey's	GAD1+/TdTom+/VGLUT1+ (PCP+METH) vs. GAD1+/VGLUT1+ (PCP+PCP)	**	0.0018	
				Tukey's	GAD1+/TdTom+/VGLUT1+ (PCP+METH) vs. TdTom+/VGLUT1+ (PCP+saline)	****	<0.0001	

				Tukey's	GAD1+/TdTom+/VGLUT1+ (PCP+METH) vs. TdTom+/VGLUT1+ (PCP+METH)	****	<0.0001	
				Tukey's	GAD1+/TdTom+/VGLUT1+ (PCP+METH) vs. TdTom+/VGLUT1+ (PCP+PCP)	****	<0.0001	
				Tukey's	GAD1+/TdTom+/VGLUT1+ (PCP+PCP) vs. GAD1+/VGLUT1+ (PCP+saline)	****	<0.0001	
				Tukey's	GAD1+/TdTom+/VGLUT1+ (PCP+PCP) vs. GAD1+/VGLUT1+ (PCP+METH)	****	<0.0001	
				Tukey's	GAD1+/TdTom+/VGLUT1+ (PCP+PCP) vs. GAD1+/VGLUT1+ (PCP+PCP)	**	0.0034	
				Tukey's	GAD1+/TdTom+/VGLUT1+ (PCP+PCP) vs. TdTom+/VGLUT1+ (PCP+saline)	****	<0.0001	
				Tukey's	GAD1+/TdTom+/VGLUT1+ (PCP+PCP) vs. TdTom+/VGLUT1+ (PCP+METH)	****	<0.0001	
				Tukey's	GAD1+/TdTom+/VGLUT1+ (PCP+PCP) vs. TdTom+/VGLUT1+ (PCP+PCP)	****	<0.0001	
				Tukey's	GAD1+/VGLUT1+ (PCP+saline) vs. GAD1+/VGLUT1+ (PCP+METH)	ns	>0.9999	
				Tukey's	GAD1+/VGLUT1+ (PCP+saline) vs. GAD1+/VGLUT1+ (PCP+PCP)	ns	0.8411	
				Tukey's	GAD1+/VGLUT1+ (PCP+saline) vs. TdTom+/VGLUT1+ (PCP+saline)	ns	0.8701	
				Tukey's	GAD1+/VGLUT1+ (PCP+saline) vs. TdTom+/VGLUT1+ (PCP+METH)	ns	0.9944	
				Tukey's	GAD1+/VGLUT1+ (PCP+saline) vs. TdTom+/VGLUT1+ (PCP+PCP)	ns	0.3984	
				Tukey's	GAD1+/VGLUT1+ (PCP+METH) vs. GAD1+/VGLUT1+ (PCP+PCP)	ns	0.9604	
				Tukey's	GAD1+/VGLUT1+ (PCP+METH) vs. TdTom+/VGLUT1+ (PCP+saline)	ns	0.5761	
				Tukey's	GAD1+/VGLUT1+ (PCP+METH) vs. TdTom+/VGLUT1+ (PCP+METH)	ns	0.9038	
				Tukey's	GAD1+/VGLUT1+ (PCP+METH) vs. TdTom+/VGLUT1+ (PCP+PCP)	ns	0.1515	
				Tukey's	GAD1+/VGLUT1+ (PCP+PCP) vs. TdTom+/VGLUT1+ (PCP+saline)	ns	0.1142	
				Tukey's	GAD1+/VGLUT1+ (PCP+PCP) vs. TdTom+/VGLUT1+ (PCP+METH)	ns	0.3026	
				Tukey's	GAD1+/VGLUT1+ (PCP+PCP) vs. TdTom+/VGLUT1+ (PCP+PCP)	*	0.0178	
				Tukey's	TdTom+/VGLUT1+ (PCP+saline) vs. TdTom+/VGLUT1+ (PCP+METH)	ns	0.9986	
				Tukey's	TdTom+/VGLUT1+ (PCP+saline) vs. TdTom+/VGLUT1+ (PCP+PCP)	ns	0.9957	
				Tukey's	TdTom+/VGLUT1+ (PCP+METH) vs. TdTom+/VGLUT1+ (PCP+PCP)	ns	0.8294	

Supplementary table 4. Statistical details of Fig. 4.

Figure	Data/Comparison	N, n/group	Primary statistic	Post-hoc test	Comparison	p value	Notation	F/t statistic
**4d**	Mean number of VGLUT1+/GAD1+ neurons.	N=24 mice, 4/group	two-way ANOVA		Interaction	<0.0001	****	F (2, 18) = 51.61
					Row Factor (effect of drug treatment)	<0.0001	****	F (2, 18) = 54.71
					Column Factor (effect of chemogenetic treatment)	<0.0001	****	F (1, 18) = 213.2
				Tukey's	saline+vehicle vs. saline+PSEM308	>0.9999	ns	
				Tukey's	saline+vehicle vs. PCP+vehicle	<0.0001	****	
				Tukey's	saline+vehicle vs. PCP+PSEM308	0.9927	ns	
				Tukey's	saline+vehicle vs. METH+vehicle	<0.0001	****	
				Tukey's	saline+vehicle vs. METH+PSEM308	0.9927	ns	
				Tukey's	saline+PSEM308 vs. PCP+vehicle	<0.0001	****	
				Tukey's	saline+PSEM308 vs. PCP+PSEM308	0.9802	ns	
				Tukey's	saline+PSEM308 vs. METH+vehicle	<0.0001	****	
				Tukey's	saline+PSEM308 vs. METH+PSEM308	0.9981	ns	
				Tukey's	PCP+vehicle vs. PCP+PSEM308	<0.0001	****	
				Tukey's	PCP+vehicle vs. METH+vehicle	0.9802	ns	
				Tukey's	PCP+vehicle vs. METH+PSEM308	<0.0001	****	
				Tukey's	PCP+PSEM308 vs. METH+vehicle	<0.0001	****	
				Tukey's	PCP+PSEM308 vs. METH+PSEM308	0.8702	ns	
				Tukey's	METH+vehicle vs. METH+PSEM308	<0.0001	****	
**4h**	Mean number of VGLUT1+/GAD1+ neurons. Between treatment groups.	N=10 mice, 5/group	Unpaired t-test		YFP vs. ChR2	<0.0001	****	t=13.31, df=8

Supplementary table 5. Statistical details of Fig. 5.

Figure	Data/Comparison	N, n/group	Primary statistic	Post-hoc test	Comparison	p value	Notation	F/t statistic
**5d**	Mean number of VGLUT1+/GAD1+ neurons. Between treatment groups.	N=25 mice, 4-5/group	two-way ANOVA		Interaction	<0.0001	****	F (2, 19) = 70.47
					Row Factor (effect of drug treatment)	<0.0001	****	F (2, 19) = 84.49
					Column Factor (effect of chemogenetic treatment)	<0.0001	****	F (1, 19) = 326.2
				Tukey's	saline+vehicle vs. saline+PSEM308	0.9204	ns	
				Tukey's	saline+vehicle vs. PCP+vehicle	<0.0001	****	
				Tukey's	saline+vehicle vs. PCP+PSEM308	0.9936	ns	
				Tukey's	saline+vehicle vs. METH+vehicle	<0.0001	****	
				Tukey's	saline+vehicle vs. METH+PSEM308	>0.9999	ns	
				Tukey's	saline+PSEM308 vs. PCP+vehicle	<0.0001	****	
				Tukey's	saline+PSEM308 vs. PCP+PSEM308	0.9982	ns	
				Tukey's	saline+PSEM308 vs. METH+vehicle	<0.0001	****	
				Tukey's	saline+PSEM308 vs. METH+PSEM308	0.9205	ns	
				Tukey's	PCP+vehicle vs. PCP+PSEM308	<0.0001	****	
				Tukey's	PCP+vehicle vs. METH+vehicle	0.9342	ns	
				Tukey's	PCP+vehicle vs. METH+PSEM308	<0.0001	****	
				Tukey's	PCP+PSEM308 vs. METH+vehicle	<0.0001	****	
				Tukey's	PCP+PSEM308 vs. METH+PSEM308	0.9924	ns	
				Tukey's	METH+vehicle vs. METH+PSEM308	<0.0001	****	
**5e**	Mean recognition index % in the NORT. Between treatment groups.	N=29 mice, 6-9/group	two-way ANOVA		Interaction	0.0002	***	F (1, 25) = 18.66
					Row Factor (effect of drug treatment)	0.0006	***	F (1, 25) = 15.42
					Column Factor (effect of chemogenetic treatment)	0.0011	**	F (1, 25) = 13.58
				Tukey's	saline+vehicle vs. saline+PSEM308	0.973	ns	
				Tukey's	saline+vehicle vs. PCP+vehicle	<0.0001	****	
				Tukey's	saline+vehicle vs. PCP+PSEM308	0.9982	ns	
				Tukey's	saline+PSEM308 vs. PCP+vehicle	<0.0001	****	
				Tukey's	saline+PSEM308 vs. PCP+PSEM308	0.9933	ns	
				Tukey's	PCP+vehicle vs. PCP+PSEM308	<0.0001	****	
**5f**	Mean alternation % in the SAT. Between treatment groups.	N=31 mice, 6-9/group	two-way ANOVA		Interaction	0.0075	**	F (1, 27) = 8.353
					Row Factor (effect of drug treatment)	0.0852	ns	F (1, 27) = 3.192
					Column Factor (effect of chemogenetic treatment)	0.0135	*	F (1, 27) = 6.994
				Tukey's	saline+vehicle vs. saline+PSEM308	0.9984	ns	
				Tukey's	saline+vehicle vs. PCP+vehicle	0.0084	**	
				Tukey's	saline+vehicle vs. PCP+PSEM308	0.9237	ns	
				Tukey's	saline+PSEM308 vs. PCP+vehicle	0.0244	*	
				Tukey's	saline+PSEM308 vs. PCP+PSEM308	0.8791	ns	
				Tukey's	PCP+vehicle vs. PCP+PSEM308	0.0017	**	
**5g**	Mean recognition index % in the NORT. Between treatment groups.	N=19 mice, 4-6/group	two-way ANOVA		Interaction	0.0005	***	F (1, 15) = 19.24
					Row Factor (effect of drug treatment)	0.0874	ns	F (1, 15) = 3.344
					Column Factor (effect of chemogenetic treatment)	0.0072	**	F (1, 15) = 9.639
				Tukey's	saline+vehicle vs. saline+PSEM308	0.8106	ns	
				Tukey's	saline+vehicle vs. METH+vehicle	0.0012	**	
				Tukey's	saline+vehicle vs. METH+PSEM308	0.8124	ns	
				Tukey's	saline+PSEM308 vs. METH+vehicle	0.0136	*	
				Tukey's	saline+PSEM308 vs. METH+PSEM308	0.3657	ns	
				Tukey's	METH+vehicle vs. METH+PSEM308	0.0004	***	
**5h**	Mean alternation % in the SAT. Between treatment groups.	N=19 mice, 4-5/group	two-way ANOVA		Interaction	0.0138	*	F (1, 15) = 7.764
					Row Factor (effect of drug treatment)	0.0572	ns	F (1, 15) = 4.242
					Column Factor (effect of chemogenetic treatment)	0.0185	*	F (1, 15) = 6.975
				Tukey's	saline+vehicle vs. saline+PSEM308	0.9996	ns	
				Tukey's	saline+vehicle vs. METH+vehicle	0.0143	*	
				Tukey's	saline+vehicle vs. METH+PSEM308	0.9735	ns	
				Tukey's	saline+PSEM308 vs. METH+vehicle	0.0257	*	
				Tukey's	saline+PSEM308 vs. METH+PSEM308	0.9579	ns	
				Tukey's	METH+vehicle vs. METH+PSEM308	0.0062	**	
**5i**	Correlation between number of VGLUT1+/GAD1+ neurons and recongition index in the NORT.	N=13 mice, 2-4/ treatment	Linear regression and Pearson's correlation analysis			0.0003	***	R squared= 0.7107
**5j**	Correlation between number of VGLUT1+/GAD1+ neurons and alternation in the SAT.	N=13 mice, 2-4/ treatment	Linear regression and Pearson's correlation analysis			0.0044	**	R squared= 0.5374
**5k**	Correlation between number of VGLUT1+/GAD1+ neurons and recongition index in the NORT.	N=12 mice, 2-4/ treatment	Linear regression and Pearson's correlation analysis			0.0001	***	R squared= 0.7823
**5l**	Correlation between number of VGLUT1+/GAD1+ neurons and alternation in the SAT.	N=12 mice, 2-4/ treatment	Linear regression and Pearson's correlation analysis			0.0003	***	R squared= 0.7524

Supplementary table 6. Statistical details of Fig. 6.

Figure	Data/Comparison	N, n/group	Primary statistic	Post-hoc test	Comparison	p value	Notation	F/t statistic
**6c**	Mean number of mCherry+/GABA+ neurons. Between treatment groups.	N=18 mice, 4-6/group	two-way ANOVA		Interaction	<0.0001	****	F (1, 14) = 74.03
					Row Factor (effect of PCP treatment)	<0.0001	****	F (1, 14) = 77.41
					Column Factor (effect of clozapine treatment)	<0.0001	****	F (1, 14) = 77.36
				Tukey's	saline+vehicle vs. saline+clozapine	0.9989	ns	
				Tukey's	saline+vehicle vs. PCP+vehicle	<0.0001	****	
				Tukey's	saline+vehicle vs. PCP+clozapine	>0.9999	ns	
				Tukey's	saline+clozapine vs. PCP+vehicle	<0.0001	****	
				Tukey's	saline+clozapine vs. PCP+clozapine	0.9991	ns	
				Tukey's	PCP+vehicle vs. PCP+clozapine	<0.0001	****	
**6e**	Mean recognition index % in the NORT. Between treatment groups.	N=27 mice, 6-7/group	two-way ANOVA		Interaction	0.0003	***	F (1, 23) = 18.40
					Row Factor (effect of PCP treatment)	0.0005	***	F (1, 23) = 16.25
					Column Factor (effect of clozapine treatment)	<0.0001	****	F (1, 23) = 30.37
				Tukey's	saline+vehicle vs. saline+clozapine	0.8313	ns	
				Tukey's	saline+vehicle vs. PCP+vehicle	<0.0001	****	
				Tukey's	saline+vehicle vs. PCP+clozapine	0.7117	ns	
				Tukey's	saline+clozapine vs. PCP+vehicle	<0.0001	****	
				Tukey's	saline+clozapine vs. PCP+clozapine	0.9979	ns	
				Tukey's	PCP+vehicle vs. PCP+clozapine	<0.0001	****	
**6f**	Mean alternation % in the SAT. Between treatment groups.	N=27 mice, 6-7/group	two-way ANOVA		Interaction	0.0781	ns	F (1, 23) = 3.399
					Row Factor (effect of PCP treatment)	0.0001	***	F (1, 23) = 21.69
					Column Factor (effect of clozapine treatment)	0.0001	***	F (1, 23) = 21.82
				Tukey's	saline+vehicle vs. saline+clozapine	0.2026	ns	
				Tukey's	saline+vehicle vs. PCP+vehicle	0.0005	***	
				Tukey's	saline+vehicle vs. PCP+clozapine	>0.9999	ns	
				Tukey's	saline+clozapine vs. PCP+vehicle	<0.0001	****	
				Tukey's	saline+clozapine vs. PCP+clozapine	0.2356	ns	
				Tukey's	PCP+vehicle vs. PCP+clozapine	0.0008	***	
**6g**	Total distance travelled after a PCP challenge. Between treatment groups.	N=31 mice, 7-8/group	two-way ANOVA		Interaction	0.6994	ns	F (1, 27) = 0.1524
					Row Factor (effect of PCP treatment)	<0.0001	****	F (1, 27) = 24.35
					Column Factor (effect of clozapine treatment)	0.0847	ns	F (1, 27) = 3.205
				Tukey's	saline+vehicle vs. saline+clozapine	0.7657	ns	
				Tukey's	saline+vehicle vs. PCP+vehicle	0.005	**	
				Tukey's	saline+vehicle vs. PCP+clozapine	0.1528	ns	
				Tukey's	saline+clozapine vs. PCP+vehicle	0.0003	***	
				Tukey's	saline+clozapine vs. PCP+clozapine	0.0146	*	
				Tukey's	PCP+vehicle vs. PCP+clozapine	0.4124	ns	

Supplementary table 7. Statistical details of Fig. 7.

Figure	Data/Comparison	N, n/group	Primary statistic	Post-hoc test	Comparison	p value	Notation	F/t statistic
**7c**	Mean number of VGLUT1+/GAD1+ neurons. Between treatment groups.	N=27 mice, 4-6/group	two-way ANOVA		Interaction	<0.0001	****	F (2, 21) = 65.43
					Row Factor (effect of drug treatment)	<0.0001	****	F (2, 21) = 86.92
					Column Factor (effect of chemogenetic treatment)	<0.0001	****	F (1, 21) = 330.4
				Tukey's	saline+vehicle vs. saline+PSEM308	0.5257	ns	
				Tukey's	saline+vehicle vs. PCP+vehicle	<0.0001	****	
				Tukey's	saline+vehicle vs. PCP+PSEM308	0.9936	ns	
				Tukey's	saline+vehicle vs. METH+vehicle	<0.0001	****	
				Tukey's	saline+vehicle vs. METH+PSEM308	0.9484	ns	
				Tukey's	saline+PSEM308 vs. PCP+vehicle	<0.0001	****	
				Tukey's	saline+PSEM308 vs. PCP+PSEM308	0.8419	ns	
				Tukey's	saline+PSEM308 vs. METH+vehicle	<0.0001	****	
				Tukey's	saline+PSEM308 vs. METH+PSEM308	0.9672	ns	
				Tukey's	PCP+vehicle vs. PCP+PSEM308	<0.0001	****	
				Tukey's	PCP+vehicle vs. METH+vehicle	0.0098	**	
				Tukey's	PCP+vehicle vs. METH+PSEM308	<0.0001	****	
				Tukey's	PCP+PSEM308 vs. METH+vehicle	<0.0001	****	
				Tukey's	PCP+PSEM308 vs. METH+PSEM308	0.9992	ns	
				Tukey's	METH+vehicle vs. METH+PSEM308	<0.0001	****	
**7d**	Mean recognition index % in the NORT. Between treatment groups.	N=23 mice, 4-7/group	two-way ANOVA		Interaction	0.0459	*	F (1, 19) = 4.563
					Row Factor (effect of drug treatment)	0.0012	**	F (1, 19) = 14.60
					Column Factor (effect of chemogenetic treatment)	0.0001	***	F (1, 19) = 23.09
				Tukey's	saline+vehicle vs. saline+PSEM308	0.3214	ns	
				Tukey's	saline+vehicle vs. PCP+vehicle	0.0011	**	
				Tukey's	saline+vehicle vs. PCP+PSEM308	0.8855	ns	
				Tukey's	saline+PSEM308 vs. PCP+vehicle	<0.0001	****	
				Tukey's	saline+PSEM308 vs. PCP+PSEM308	0.6866	ns	
				Tukey's	PCP+vehicle vs. PCP+PSEM308	0.0002	***	
**7e**	Mean alternation % in the SAT. Between treatment groups.	N=25 mice, 4-8/group	two-way ANOVA		Interaction	0.0874	ns	F (1, 21) = 3.215
					Row Factor (effect of drug treatment)	0.0282	*	F (1, 21) = 5.560
					Column Factor (effect of chemogenetic treatment)	0.0138	*	F (1, 21) = 7.226
				Tukey's	saline+vehicle vs. saline+PSEM308	0.9388	ns	
				Tukey's	saline+vehicle vs. PCP+vehicle	0.0216	*	
				Tukey's	saline+vehicle vs. PCP+PSEM308	0.9946	ns	
				Tukey's	saline+PSEM308 vs. PCP+vehicle	0.013	*	
				Tukey's	saline+PSEM308 vs. PCP+PSEM308	0.9818	ns	
				Tukey's	PCP+vehicle vs. PCP+PSEM308	0.0088	**	
**7f**	Total distance travelled after a PCP challenge. Between treatment groups.	N=25 mice, 4-8/group	two-way ANOVA		Interaction	0.0199	*	F (1, 21) = 6.351
					Row Factor (effect of drug treatment)	0.0054	**	F (1, 21) = 9.627
					Column Factor (effect of chemogenetic treatment)	0.0494	*	F (1, 21) = 4.350
				Tukey's	saline+vehicle vs. saline+PSEM308	0.9915	ns	
				Tukey's	saline+vehicle vs. PCP+vehicle	0.0011	**	
				Tukey's	saline+vehicle vs. PCP+PSEM308	0.873	ns	
				Tukey's	saline+PSEM308 vs. PCP+vehicle	0.0105	*	
				Tukey's	saline+PSEM308 vs. PCP+PSEM308	0.9818	ns	
				Tukey's	PCP+vehicle vs. PCP+PSEM308	0.0099	**	
**7g**	Mean recognition index % in the NORT. Between treatment groups.	N=32 mice, 7-9/group	two-way ANOVA		Interaction	0.0009	***	F (1, 28) = 13.91
					Row Factor (effect of drug treatment)	<0.0001	****	F (1, 28) = 24.97
					Column Factor (effect of chemogenetic treatment)	<0.0001	****	F (1, 28) = 24.15
				Tukey's	saline+vehicle vs. saline+PSEM308	0.8482	ns	
				Tukey's	saline+vehicle vs. METH+vehicle	<0.0001	****	
				Tukey's	saline+vehicle vs. METH+PSEM308	>0.9999	ns	
				Tukey's	saline+PSEM308 vs. METH+vehicle	<0.0001	****	
				Tukey's	saline+PSEM308 vs. METH+PSEM308	0.8205	ns	
				Tukey's	METH+vehicle vs. METH+PSEM308	<0.0001	****	
**7h**	Mean alternation % in the SAT. Between treatment groups.	N=34 mice, 8-9/group	two-way ANOVA		Interaction	0.2425	ns	F (1, 30) = 1.422
					Row Factor (effect of drug treatment)	0.001	***	F (1, 30) = 13.31
					Column Factor (effect of chemogenetic treatment)	0.0019	**	F (1, 30) = 11.60
				Tukey's	saline+vehicle vs. saline+PSEM308	0.4132	ns	
				Tukey's	saline+vehicle vs. METH+vehicle	0.0093	**	
				Tukey's	saline+vehicle vs. METH+PSEM308	0.998	ns	
				Tukey's	saline+PSEM308 vs. METH+vehicle	0.0002	***	
				Tukey's	saline+PSEM308 vs. METH+PSEM308	0.3234	ns	
				Tukey's	METH+vehicle vs. METH+PSEM308	0.0143	*	

**7i**	Total distance travelled after a METH challenge. Between treatment groups.	N=34 mice, 7-9/group	two-way ANOVA		Interaction	0.0263	*	F (1, 30) = 5.465
					Row Factor (effect of drug treatment)	<0.0001	****	F (1, 30) = 40.95
					Column Factor (effect of chemogenetic treatment)	0.0841	ns	F (1, 30) = 3.192
				Tukey's	saline+vehicle vs. saline+PSEM308	0.9814	ns	
				Tukey's	saline+vehicle vs. METH+vehicle	<0.0001	****	
				Tukey's	saline+vehicle vs. METH+PSEM308	0.0105	*	
				Tukey's	saline+PSEM308 vs. METH+vehicle	<0.0001	****	
				Tukey's	saline+PSEM308 vs. METH+PSEM308	0.0436	*	
				Tukey's	METH+vehicle vs. METH+PSEM308	0.0251	*	

Supplementary table 8. Statistical details of Supplementary Fig. 1

Supplementary Fig.	Data/Comparison	N, n/group	Primary statistic	Post-hoc test	Comparison	p value	Notation	F/t statistic
**S1c (left)**	Mean of mCherry+/GABA+ expressed as % of total	N=10 mice, 5/group	Unpaired t-test		saline vs. PCP	<0.0001	****	t=7.379, df=8
**S1c (right)**	Mean of mCherry+GAD67+ expressed as % of total	N=10 mice, 5/group	Unpaired t-test		saline vs. PCP	<0.0001	****	t=8.594, df=8
**S1d**	Mean number of PL mCherry+ neurons. Between treatment	N=10 mice, 5/group	Unpaired t-test		saline vs. PCP	0.7592	ns	t=0.3172, df=8
**S1e**	Mean number of PL GABA+/mCherry- neurons.	N=10 mice, 5/group	Unpaired t-test		saline vs. PCP	0.5926	ns	t=0.5573, df=8

Supplementary table 9. Statistical details of Supplementary Fig. 2

Supplementary Fig.	Data/Comparison	N, n/group	Primary statistic	Post-hoc test	Comparison	p value	Notation	F/t statistic
**S2d**	Mean number of ki67+ cells per mm2. Between treatment groups.	N=12 mice, 3/group	one-way ANOVA		Mean effect of group	<0.0001	****	F (3, 8) = 80.56
				Tukey's	DG vs. PL-saline	<0.0001	****	
				Tukey's	DG vs. PL-PCP	<0.0001	****	
				Tukey's	DG vs. PL-METH	<0.0001	****	
				Tukey's	PL-saline vs. PL-PCP	>0.9999	ns	
				Tukey's	PL-saline vs. PL-METH	>0.9999	ns	
				Tukey's	PL-PCP vs. PL-METH	>0.9999	ns	
**S2e**	Mean number of DCX+ cells per mm2. Between treatment groups.	N=12 mice, 3/group	one-way ANOVA		Mean effect of group	<0.0001	****	F (3, 8) = 312.4
				Tukey's	DG vs. PL-saline	0.0001	***	
				Tukey's	DG vs. PL-PCP	0.0001	***	
				Tukey's	DG vs. PL-METH	0.0001	***	
				Tukey's	PL-saline vs. PL-PCP	>0.9999	ns	
				Tukey's	PL-saline vs. PL-METH	>0.9999	ns	
				Tukey's	PL-PCP vs. PL-METH	>0.9999	ns	
**S2f**	Mean number of TUNEL+ cells per mm2. Between treatment groups.	N=12 mice, 3/group	one-way ANOVA		Mean effect of group	<0.0001	****	F (3, 8) = 312.4
				Tukey's	DNase vs. saline	<0.0001	****	
				Tukey's	DNase vs. METH	<0.0001	****	
				Tukey's	DNase vs. PCP	<0.0001	****	
				Tukey's	saline vs. METH	0.9994	ns	
				Tukey's	saline vs. PCP	0.9996	ns	
				Tukey's	METH vs. PCP	0.9963	ns	

Supplementary table 10. Statistical details of Supplementary Fig. 3

Supplementary Fig.	Data/Comparison	N, n/group	Primary statistic	Post-hoc test	Comparison	p value	Notation	F/t statistic
**S3d (left)**	GAD1-/mCherry+/VGLUT1- and GAD1+/mCherry+/VGLUT1- neurons expressed as % of total mCherry+ neurons in saline-treated mice. Comparison between types of neurons.	N=6 mice, 3/group	Unpaired t-test		GAD1-/mCherry+/VGLUT1- vs. GAD1+/mCherry+/VGLUT1-	0.0248	*	t=3.504, df=4
**S3d (center)**	GAD1-/mCherry+/VGLUT1- and GAD1+/mCherry+/VGLUT1- neurons expressed as % of total mCherry+ neurons in PCP-treated mice. Comparison between types of neurons.	N=6 mice, 3/group	Unpaired t-test		GAD1-/mCherry+/VGLUT1- vs. GAD1+/mCherry+/VGLUT1-	0.0632	ns	t=2.551, df=4
**S3d (right)**	GAD1-/mCherry+/VGLUT1- and GAD1+/mCherry+/VGLUT1- neurons expressed as % of total mCherry+ neurons in METH-treated mice. Comparison between types of neurons.	N=6 mice, 3/group	Unpaired t-test		GAD1-/mCherry+/VGLUT1- vs. GAD1+/mCherry+/VGLUT1-	0.0368	*	t=3.082, df=4
**S3e (left)**	GAD1+/mCherry+/VGLUT1+, GAD1+/VGLUT1+ and GAD1+/mCherry+ neurons expressed as % of total neurons counted in saline-treated mice. Comparison between types of neurons.	N=6 mice, 3/group	one-way ANOVA		Mean effect of cell type	0.0015	**	F (2, 6) = 23.35
				Tukey's	mCherry+/VGLUT1+/GAD1+ vs. VGLUT1+/GAD1+	0.0026	**	
				Tukey's	mCherry+/VGLUT1+/GAD1+ vs. mCherry+/GAD1+	0.0025	**	
				Tukey's	VGLUT1+/GAD1+ vs. mCherry+/GAD1+	0.9984	ns	
**S3e (center)**	GAD1+/mCherry+/VGLUT1+, GAD1+/VGLUT1+ and GAD1+/mCherry+ neurons expressed as % of total neurons counted in PCP-treated mice. Comparison between types of neurons.	N=6 mice, 3/group	one-way ANOVA		Mean effect of cell type	<0.0001	****	F (2, 6) = 492.4
				Tukey's	mCherry+/VGLUT1+/GAD1+ vs. VGLUT1+/GAD1+	<0.0001	****	
				Tukey's	mCherry+/VGLUT1+/GAD1+ vs. mCherry+/GAD1+	<0.0001	****	
				Tukey's	VGLUT1+/GAD1+ vs. mCherry+/GAD1+	0.0216	*	
**S3e (right)**	GAD1+/mCherry+/VGLUT1+, GAD1+/VGLUT1+ and GAD1+/mCherry+ neurons expressed as % of total neurons counted in METH-treated mice. Comparison between types of neurons.	N=6 mice, 3/group	one-way ANOVA		Mean effect of cell type	<0.0001	****	F (2, 6) = 232.0
				Tukey's	mCherry+/VGLUT1+/GAD1+ vs. VGLUT1+/GAD1+	<0.0001	****	
				Tukey's	mCherry+/VGLUT1+/GAD1+ vs. mCherry+/GAD1+	<0.0001	****	
				Tukey's	VGLUT1+/GAD1+ vs. mCherry+/GAD1+	0.1	ns	

Supplementary table 11. Statistical details of Supplementary Fig. 4

Supplementary Fig.	Data/Comparison	N, n/group	Primary statistic	Post-hoc test	Comparison	p value	Notation	F/t statistic
**S4b**	Percentage of cell area occupied by VGAT RNA puncta (3 mice, 25 cells per cell type/mouse). Between groups of different neuronal types in saline-treated mice.	N=3 mice, 3/group	Kruskal-Wallis test		Mean effect of group	<0.0001	****	Kruskal-Wallis statistic: 149.9
				Dunn's	mCherry+ only vs. mCherry+/GAD1+	<0.0001	****	
				Dunn's	mCherry+ only vs. GAD1+ only	<0.0001	****	
				Dunn's	mCherry+/GAD1+ vs. GAD1+ only	0.956	ns	
**S4c**	Percentage of cell area occupied by VGLUT1 RNA puncta (3 mice, 25 cells per cell type/mouse). Between groups of different neuronal types in saline-treated mice.	N=3 mice, 3/group	Kruskal-Wallis test		Mean effect of group	<0.0001	****	Kruskal-Wallis statistic: 161.5
				Dunn's	mCherry+ only vs. mCherry+/GAD1+	<0.0001	****	
				Dunn's	mCherry+ only vs. GAD1+ only	<0.0001	****	
				Dunn's	mCherry+/GAD1+ vs. GAD1+ only	<0.0001	****	
**S4d**	Percentage of cell area occupied by VGAT RNA puncta in mCherry+/GAD1+. Between treatment groups.	N=3 mice, 3/group	Kruskal-Wallis test		Mean effect of treatment	0.2308	ns	Kruskal-Wallis statistic: 2.933
				Dunn's	saline vs. PCP	>0.9999	ns	
				Dunn's	saline vs. METH	0.3018	ns	
				Dunn's	PCP vs. METH	0.6423	ns	
**S4e**	Percentage of cell area occupied by VGLUT1 RNA puncta in mCherry+/GAD1+. Between treatment groups.	N=3 mice, 3/group	Kruskal-Wallis test		Mean effect of treatment	0.0896	ns	Kruskal-Wallis statistic: 4.824
				Dunn's	saline vs. PCP	0.1714	ns	
				Dunn's	saline vs. METH	>0.9999	ns	
				Dunn's	PCP vs. METH	0.1714	ns	

Supplementary table 12. Statistical details of Supplementary Fig. 5

Supplementary Fig.	Data/Comparison	N, n/group	Primary statistic	Post-hoc test	Comparison	p value	Notation	F/t statistic
**S5c**	Mean number of PL GAD67+/TdTomato+ neurons. Between treatment groups.	N=8 mice, 4/group	Unpaired t-test		saline vs. PCP	0.0334	*	t=2.747, df=6
**S5d**	Mean of GAD67+/TdTomato+ expressed as % of total TdTomato+. Between treatment groups.	N=8 mice, 4/group	Unpaired t-test		saline vs. PCP	0.0087	**	t=3.821, df=6
**S5e**	Mean number of PL TdTomato+ neurons. Between treatment groups.	N=8 mice, 4/group	Unpaired t-test		saline vs. PCP	0.2518	ns	t=1.268, df=6

Supplementary table 13. Statistical details of Supplementary Fig. 6

Supplementary Fig.	Data/Comparison	N, n/group	Primary statistic	Post-hoc test	Comparison	p value	Notation	F/t statistic
S6c	Mean number of GFP+/GABA+ neurons expressed as % of total GFP+ cells. Between treatment groups.	N=14 mice, 3-4/group	two-way ANOVA		Interaction	<0.0001	****	F (1, 10) = 40.03
					Row Factor (effect of drug treatment)	<0.0001	****	F (1, 10) = 40.04
					Column Factor (effect of AAV)	<0.0001	****	F (1, 10) = 221.9
				Tukey's	saline/AAV-shScr vs. saline/AAV-shGAD1	0.0007	***	
				Tukey's	saline/AAV-shScr vs. PCP/AAV-shScr	<0.0001	****	
				Tukey's	saline/AAV-shScr vs. PCP/AAV-shGAD1	0.0004	***	
				Tukey's	saline/AAV-shGAD1 vs. PCP/AAV-shScr	<0.0001	****	
				Tukey's	saline/AAV-shGAD1 vs. PCP/AAV-shGAD1	>0.9999	ns	
				Tukey's	PCP/AAV-shScr vs. PCP/AAV-shGAD1	<0.0001	****	
S6d	Total distance travelled after the first and the last of 10 PCP injections. Between treatment & time groups.	N=38 mice, 8-10/group	two-way ANOVA		Interaction	0.0001	***	F (3, 68) = 12.25
					Row Factor (effect of drug treatment)	<0.0001	****	F (3, 68) = 156.0
					Column Factor (effect of AAV)	<0.0001	****	F (1, 68) = 22.90
				Tukey's	saline/AAV-shScr (DAY1) vs. saline/AAV-shScr (DAY10)	0.9997	ns	
				Tukey's	saline/AAV-shScr (DAY1) vs. saline/AAV-shGAD1 (DAY1)	0.995	ns	
				Tukey's	saline/AAV-shScr (DAY1) vs. saline/AAV-shGAD1 (DAY10)	0.9422	ns	
				Tukey's	saline/AAV-shScr (DAY1) vs. PCP/AAV-shScr (DAY1)	<0.0001	****	
				Tukey's	saline/AAV-shScr (DAY1) vs. PCP/AAV-shScr (DAY10)	<0.0001	****	
				Tukey's	saline/AAV-shScr (DAY1) vs. PCP/AAV-shGAD1 (DAY1)	<0.0001	****	
				Tukey's	saline/AAV-shScr (DAY1) vs. PCP/AAV-shGAD1 (DAY10)	<0.0001	****	
				Tukey's	saline/AAV-shScr (DAY10) vs. saline/AAV-shGAD1 (DAY1)	>0.9999	ns	
				Tukey's	saline/AAV-shScr (DAY10) vs. saline/AAV-shGAD1(DAY1)	0.9973	ns	
				Tukey's	saline/AAV-shScr (DAY10) vs. PCP/AAV-shScr (DAY1)	<0.0001	****	
				Tukey's	saline/AAV-shScr (DAY10) vs. PCP/AAV-shScr (DAY10)	<0.0001	****	
				Tukey's	saline/AAV-shScr :DAY10 vs. PCP/AAV-shGAD1 (DAY1)	<0.0001	****	
				Tukey's	saline/AAV-shScr (DAY10) vs. PCP/AAV-shGAD1 (DAY10)	<0.0001	****	
				Tukey's	saline/AAV-shGAD1 (DAY1) vs. saline/AAV-shGAD1 (DAY10)	>0.9999	ns	
				Tukey's	saline/AAV-shGAD1(DAY1) vs. PCP/AAV-shScr (DAY1)	<0.0001	****	
				Tukey's	saline/AAV-shGAD1 (DAY1) vs. PCP/AAV-shScr :DAY10	<0.0001	****	
				Tukey's	saline/AAV-shGAD1 (DAY1) vs. PCP/AAV-shGAD1 (DAY1)	<0.0001	****	
				Tukey's	saline/AAV-shGAD1 (DAY1) vs. PCP/AAV-shGAD1 (DAY10)	<0.0001	****	
				Tukey's	saline/AAV-shGAD1 (DAY10) vs. PCP/AAV-shScr (DAY1)	<0.0001	****	
				Tukey's	saline/AAV-shGAD1 (DAY10) vs. PCP/AAV-shScr (DAY10)	<0.0001	****	
				Tukey's	saline/AAV-shGAD1 (DAY10) vs. PCP/AAV-shGAD1 (DAY1)	<0.0001	****	
				Tukey's	saline/AAV-shGAD1 (DAY10) vs. PCP/AAV-shGAD1 (DAY10)	<0.0001	****	
				Tukey's	PCP/AAV-shScr (DAY1) vs. PCP/AAV-shScr (DAY10)	<0.0001	****	
				Tukey's	PCP/AAV-shScr (DAY1) vs. PCP/AAV-shGAD1 (DAY1)	>0.9999	ns	
				Tukey's	PCP/AAV-shScr (DAY1) vs. PCP/AAV-shGAD1 (DAY10)	0.9951	ns	
				Tukey's	PCP/AAV-shScr (DAY10) vs. PCP/AAV-shGAD1 (DAY1)	<0.0001	****	
				Tukey's	PCP/AAV-shScr (DAY10) vs. PCP/AAV-shGAD1 (DAY10)	<0.0001	****	
				Tukey's	PCP/AAV-shGAD1 (DAY1) vs. PCP/AAV-shGAD1 (DAY10)	0.9922	ns	

**S6e**	Time to criterion in the NORT. Between treatment groups.	N=31 mice, 6-9/group	two-way ANOVA		Interaction	0.8399	ns	F (1, 27) = 0.0416
					Row Factor (effect of drug treatment)	0.3868	ns	F (1, 27) = 0.7737
					Column Factor (effect of AAV)	0.2223	ns	F (1, 27) = 1.561
				Tukey's	saline/AAV-shScr vs. saline/AAV-shGAD1	0.7186	ns	
				Tukey's	saline/AAV-shScr vs. PCP/AAV-shScr	0.9682	ns	
				Tukey's	saline/AAV-shScr vs. PCP/AAV-shGAD1	0.9925	ns	
				Tukey's	saline/AAV-shGAD1 vs. PCP/AAV-shScr	0.4912	ns	
				Tukey's	saline/AAV-shGAD1 vs. PCP/AAV-shGAD1	0.8492	ns	
				Tukey's	PCP/AAV-shScr vs. PCP/AAV-shGAD1	0.8881	ns	
**S6f**	Total number of entries in the SAT. Between treatment groups.	N=34 mice, 6-10/group	ART factorial ANOVA		Interaction	0.9757	ns	F (1, 29) = 0.023
					Row Factor (effect of drug treatment)	0.8791	ns	F (1, 29) = 0.001
					Column Factor (effect of AAV)	0.86755	ns	F (1, 29) = 0.028
				Mann Whitney + Bonferroni correction	saline/AAV-shScr vs. saline/AAV-shGAD1	1.9452	ns	
					saline/AAV-shScr vs. PCP/AAV-shScr	2.8488	ns	
					saline/AAV-shScr vs. PCP/AAV-shGAD1	2.1672	ns	
					saline/AAV-shGAD1 vs. PCP/AAV-shScr	2.9928	ns	
					saline/AAV-shGAD1 vs. PCP/AAV-shGAD1	2.8308	ns	
					PCP/AAV-shScr vs. PCP/AAV-shGAD1	4.8744	ns	

Supplementary table 14. Statistical details of Supplementary Fig. 7

Supplementary Fig.	Data/Comparison	N, n/group	Primary statistic	Post-hoc test	Comparison	p value	Notation	F/t statistic
**S7c**	Mean number of GFP+/GABA+ neurons expressed as % of total GFP+ cells. Between treatment groups.	N=8 mice, 4/group	Unpaired t-test		AAV-shScr vs. AAV-shScr	<0.0001	****	t=15.92, df=6
**S7d**	Mean recognition index % in the NORT. Between treatment groups.	N=17 mice, 8-9/group	Unpaired t-test		AAV-shScr vs. AAV-shScr	0.6281	ns	t=0.4945, df=15
**S7e**	Time to criterion in the NORT. Between treatment groups.	N=17 mice, 8-9/group	Unpaired t-test		AAV-shScr vs. AAV-shScr	0.2754	ns	t=1.132, df=15
**S7f**	Mean alternation % in the SAT. Between treatment groups.	N=16 mice, 8/group	Unpaired t-test		AAV-shScr vs. AAV-shScr	0.2452	ns	t=1.213, df=14
**S7g**	Total number of entries in the SAT. Between treatment groups.	N=16 mice, 8/group	Unpaired t-test		AAV-shScr vs. AAV-shScr	0.0711	ns	t=1.953, df=14

Supplementary table 15. Statistical details of Supplementary Fig. 8

Supplementary Fig.	Data/Comparison	N, n/group	Primary statistic	Post-hoc test	Comparison	p value	Notation	F/t statistic
**S8a (left)**	Mean of mCherry+/GABA+ expressed as % of total mCherry+. Between treatment groups.	N=10 mice, 5/group	Unpaired t-test		saline vs. METH	0.0065	**	t=3.651, df=8
**S8a (right)**	Mean of mCherry+GAD67+ expressed as % of total mCherry+. Between treatment groups.	N=10 mice, 5/group	Unpaired t-test		saline vs. METH	0.0038	**	t=4.035, df=8
**S8b (left)**	Mean number of PL mCherry+ neurons. Between treatment groups.	N=10 mice, 5/group	Unpaired t-test		saline vs. METH	0.3983	ns	t=0.8923, df=8
**S8b (right)**	Mean number of PL GABA+/mCherry- neurons. Between treatment groups.	N=6 mice, 3/group	Unpaired t-test		saline vs. METH	0.8129	ns	t=0.2527, df=4

Supplementary table 16. Statistical details of Supplementary Fig. 9

Supplementary Fig.	Data/Comparison	N, n/group	Primary statistic	Post-hoc test	Comparison	p value	Notation	F/t statistic
**S9e**	Mean number of FG+/mCherry+/GABA+ neurons in total FG+/mCherry+ neurons. Between treatment groups.	N=10 mice, 3-4/group	one-way ANOVA		Mean effect of group	0.0065	**	F (2, 7) = 11.27
				Tukey's	saline vs. PCP	0.0196	*	
				Tukey's	saline vs. METH	0.0067	**	
				Tukey's	PCP vs. METH	0.8153	ns	

Supplementary table 17. Statistical details of Supplementary Fig. 11

Supplementary Fig.	Data/Comparison	N, n/group	Primary statistic	Post-hoc test	Comparison	p value	Notation	F/t statistic
**S11f**	Mean fraction of TH+ neurons co-expressing c-fos after acute treatment. Between treatment groups.	N=22 mice, 3-4/group	two-way ANOVA		Interaction	0.0053	**	F (2, 16) = 7.411
					Row Factor (effect of PCP/METH treatment)	0.0042	**	F (2, 16) = 7.846
					Column Factor (effect of PSEM)	0.001	***	F (1, 16) = 16.15
				Tukey's	saline+vehicle vs. saline+PSEM308	0.9572	ns	
				Tukey's	saline+vehicle vs. PCP+vehicle	0.0014	**	
				Tukey's	saline+vehicle vs. PCP+PSEM308	0.8571	ns	
				Tukey's	saline+vehicle vs. METH+vehicle	0.0197	*	
				Tukey's	saline+vehicle vs. METH+PSEM308	0.9998	ns	
				Tukey's	saline+PSEM308 vs. PCP+vehicle	0.0036	**	
				Tukey's	saline+PSEM308 vs. PCP+PSEM308	0.9993	ns	
				Tukey's	saline+PSEM308 vs. METH+vehicle	0.0597	ns	
				Tukey's	saline+PSEM308 vs. METH+PSEM308	0.9876	ns	
				Tukey's	PCP+vehicle vs. PCP+PSEM308	0.0069	**	
				Tukey's	PCP+vehicle vs. METH+vehicle	0.874	ns	
				Tukey's	PCP+vehicle vs. METH+PSEM308	0.001	**	
				Tukey's	PCP+PSEM308 vs. METH+vehicle	0.1054	ns	
				Tukey's	PCP+PSEM308 vs. METH+PSEM308	0.9246	ns	
				Tukey's	METH+vehicle vs. METH+PSEM308	0.0197	*	

Supplementary table 18. Statistical details of Supplementary Fig. 12

Supplementary Fig.	Data/Comparison	N, n/group	Primary statistic	Post-hoc test	Comparison	p value	Notation	F/t statistic
**S12f**	Mean fraction of TH+ neurons co-expressing c-fos after acute stimulation. Between treatment groups.	N=8 mice, 4/group	Unpaired t-test		YFP vs. ChR2	0.0001	***	t=8.911, df=6
**S12h**	Mean number of c-fos+ neurons after acute stimulation. Between treatment groups.	N=9 mice, 4-5/group	Unpaired t-test		YFP vs. ChR2	<0.0001	****	t=12.23, df=7
**S4O**	Mean number of c-fos+/GABA- and c-fos+/GABA+ neurons after acute stimulation. Between treatment groups.	N=9 mice, 4-5/group	two-way ANOVA	Within each column, compare rows	Interaction	<0.0001	****	F (1, 14) = 108.7
					Row Factor (effect of treatment)	<0.0001	****	F (1, 14) = 483.4
					Column Factor (GABA+ or GABA- cells)	<0.0001	****	F (1, 14) = 114.0
				Tukey's	YFP vs. ChR2 (GABA-/c-fos+)	<0.0001	****	
				Tukey's	YFP vs. ChR2 (GABA+/c-fos+)	0.9979	ns	
**S12k**	Mean of VGLUT1+/GAD1+ expressed as % of total VGLUT1+. Between treatment groups.	N=10 mice, 5/group	Unpaired t-test		YFP vs. ChR2	<0.0001	****	t=9.292, df=8
**S12l**	Mean number of PL VGLUT1+ neurons. Between treatment groups.	N=10 mice, 5/group	Unpaired t-test		YFP vs. ChR2	0.6131	ns	t=0.5260, df=8

Supplementary table 19. Statistical details of Supplementary Fig. 13

	Data/Comparison	N, n/group	Primary statistic	Post-hoc test	Comparison	p value	Notation	F/t statistic
**S13d**	Mean of VGLUT1+/GAD1+ neurons. Between treatment groups.	N= 6 mice, 3/group	Unpaired t-test		YFP vs. ChR2	0.2181	ns	t=1.460, df=4

Supplementary table 20. Statistical details of Supplementary Fig. 14

Supplementary Fig.	Data/Comparison	N, n/group	Primary statistic	Post-hoc test	Comparison	p value	Notation	F/t statistic
**S14c**	Mean number of c-fos+ neurons after 1 acute drug/saline injection. Between treatment groups.	N=12, 4/group	one-way ANOVA		Mean effect of group	<0.0001	****	F (2, 9) = 41.22
				Dunnett's	saline vs. PCP	<0.0001	****	
				Dunnett's	saline vs. METH	<0.0001	****	
**S14d**	Mean number of c-fos+/mCherry+ and c-fos+/mCherry- neurons after 1 acute drug/saline injection. Between treatment groups.	N=12, 4/group	two-way ANOVA	Within each column, compare rows	Interaction	<0.0001	****	F (2, 18) = 35.54
					Row Factor (effect of treatment)	<0.0001	****	F (2, 18) = 44.31
					Column Factor (mCherry+ or mCherry- cells)	<0.0001	****	F (1, 18) = 204.6
				Tukey's	saline vs. PCP (c-fos+/mCherry+)	<0.0001	****	
				Tukey's	saline vs. METH (c-fos+/mCherry+)	<0.0001	****	
				Tukey's	PCP vs. METH (c-fos+/mCherry+)	0.4371	ns	
				Tukey's	saline vs. PCP (c-fos+/mCherry-)	0.7516	ns	
				Tukey's	saline vs. METH (c-fos+/mCherry-)	0.9024	ns	
				Tukey's	PCP vs. METH (c-fos+/mCherry-)	0.9539	ns	
**S14e**	Mean fraction of PV+ neurons co-expressing c-fos after 1 acute drug/saline injection. Between treatment groups.	N=12, 4/group	one-way ANOVA		Mean effect of group	0.7577	ns	F (2, 9) = 0.2861
				Dunnett's	saline vs. PCP	0.9076	ns	
				Dunnett's	saline vs. METH	0.6799	ns	
**S14f**	Mean number of c-fos+ neurons after the last of 10 drug/saline injections. Between treatment groups.	N=12, 4/group	one-way ANOVA		Mean effect of group	0.0004	***	F (2, 9) = 20.69
				Dunnett's	saline vs. PCP	0.0056	**	
				Dunnett's	saline vs. METH	0.0003	***	
**S14g**	Mean number of c-fos+/mCherry+ and c-fos+/mCherry- neurons after the last of 10 drug/saline injections. Between treatment groups.	N=12, 4/group	two-way ANOVA	Within each column, compare rows	Interaction	0.0003	***	F (2, 18) = 13.06
					Row Factor (effect of treatment)	<0.0001	****	F (2, 18) = 19.79
					Column Factor (mCherry+ or mCherry- cells)	<0.0001	****	F (1, 18) = 90.20
				Tukey's	saline vs. PCP (c-fos+/mCherry+)	0.0004	***	
				Tukey's	saline vs. METH (c-fos+/mCherry+)	<0.0001	****	
				Tukey's	PCP vs. METH (c-fos+/mCherry+)	0.0131	*	
				Tukey's	saline vs. PCP (c-fos+/mCherry-)	0.7413	ns	
				Tukey's	saline vs. METH (c-fos+/mCherry-)	0.7107	ns	
				Tukey's	PCP vs. METH (c-fos+/mCherry-)	0.9985	ns	
**S14h**	Mean fraction of PV+ neurons co-expressing c-fos after the last of 10 drug/saline injections. Between treatment	N=12, 4/group	one-way ANOVA		Mean effect of group	0.0922	ns	F (2, 9) = 3.144
				Dunnett's	saline vs. PCP	0.9869	ns	
				Dunnett's	saline vs. METH	0.0913	ns	

Supplementary table 21. Statistical details of Supplementary Fig. 15

Supplementary Fig.	Data/Comparison	N, n/group	Primary statistic	Post-hoc test	Comparison	p value	Notation	F/t statistic
**S15f**	Mean number of c-fos+/GFP+ neurons in total GFP+ neurons. Between treatment groups.	N=18 mice, 3/group	two-way ANOVA		Interaction	0.4269	ns	F (2, 12) = 0.9147
					Row Factor (effect of drug treatment)	0.6483	ns	F (2, 12) = 0.4495
					Column Factor (effect of chemogenetic activation)	<0.0001	****	F (1, 12) = 72.46
				Tukey's	saline+vehicle vs. saline+PSEM308	0.0053	**	
				Tukey's	saline+vehicle vs. PCP+vehicle	0.9393	ns	
				Tukey's	saline+vehicle vs. PCP+PSEM308	0.0033	**	
				Tukey's	saline+vehicle vs. METH+vehicle	>0.9999	ns	
				Tukey's	saline+vehicle vs. METH+PSEM308	0.0008	***	
				Tukey's	saline+PSEM308 vs. PCP+vehicle	0.0241	*	
				Tukey's	saline+PSEM308 vs. PCP+PSEM308	0.9996	ns	
				Tukey's	saline+PSEM308 vs. METH+vehicle	0.0047	**	
				Tukey's	saline+PSEM308 vs. METH+PSEM308	0.8303	ns	
				Tukey's	PCP+vehicle vs. PCP+PSEM308	0.0146	*	
				Tukey's	PCP+vehicle vs. METH+vehicle	0.9182	ns	
				Tukey's	PCP+vehicle vs. METH+PSEM308	0.0033	**	
				Tukey's	PCP+PSEM308 vs. METH+vehicle	0.0029	**	
				Tukey's	PCP+PSEM308 vs. METH+PSEM308	0.9389	ns	
				Tukey's	METH+vehicle vs. METH+PSEM308	0.0007	***	
**S15h**	Mean number of c-fos+/GFP- neurons. Between treatment groups.	N=18 mice, 3/group	two-way ANOVA		Interaction	0.0007	***	F (2, 12) = 14.17
					Row Factor (effect of drug treatment)	0.0005	***	F (2, 12) = 15.04
					Column Factor (effect of chemogenetic treatment)	<0.0001	****	F (1, 12) = 48.55
				Tukey's	saline+vehicle vs. saline+PSEM308	0.9996	ns	
				Tukey's	saline+vehicle vs. PCP+vehicle	0.0002	***	
				Tukey's	saline+vehicle vs. PCP+PSEM308	0.9999	ns	
				Tukey's	saline+vehicle vs. METH+vehicle	0.0004	***	
				Tukey's	saline+vehicle vs. METH+PSEM308	0.9885	ns	
				Tukey's	saline+PSEM308 vs. PCP+vehicle	0.0003	***	
				Tukey's	saline+PSEM308 vs. PCP+PSEM308	>0.9999	ns	
				Tukey's	saline+PSEM308 vs. METH+vehicle	0.0006	***	
				Tukey's	saline+PSEM308 vs. METH+PSEM308	0.9995	ns	
				Tukey's	PCP+vehicle vs. PCP+PSEM308	0.0003	***	
				Tukey's	PCP+vehicle vs. METH+vehicle	0.9978	ns	
				Tukey's	PCP+vehicle vs. METH+PSEM308	0.0005	***	
				Tukey's	PCP+PSEM308 vs. METH+vehicle	0.0005	***	
				Tukey's	PCP+PSEM308 vs. METH+PSEM308	0.9987	ns	
				Tukey's	METH+vehicle vs. METH+PSEM308	0.0009	***	
**S15i**	Mean number of c-fos+/GABA+ and c-fos+/GABA- neurons after injections. Between treatment groups.	N=18 mice, 3/group	two-way ANOVA		Interaction	<0.0001	****	F (5, 24) = 14.61
					Row Factor (effect of treatment)	<0.0001	****	F (5, 24) = 16.84
					Column Factor (GABA+ or GABA- cells)	<0.0001	****	F (1, 24) = 318.7
				Tukey's	saline+vehicle vs. saline+PSEM308 (GABA-/c-fos+)	0.655	ns	
				Tukey's	saline+vehicle vs. PCP+vehicle (GABA-/c-fos+)	<0.0001	****	
				Tukey's	saline+vehicle vs. PCP+PSEM308 (GABA-/c-fos+)	0.9995	ns	
				Tukey's	saline+vehicle vs. METH+vehicle (GABA-/c-fos+)	<0.0001	****	
				Tukey's	saline+vehicle vs. METH+PSEM308 (GABA-/c-fos+)	>0.9999	ns	
				Tukey's	saline+PSEM308 vs. PCP+vehicle (GABA-/c-fos+)	<0.0001	****	
				Tukey's	saline+PSEM308 vs. PCP+PSEM308 (GABA-/c-fos+)	0.8346	ns	
				Tukey's	saline+PSEM308 vs. METH+vehicle (GABA-/c-fos+)	<0.0001	****	
				Tukey's	saline+PSEM308 vs. METH+PSEM308 (GABA-/c-fos+)	0.7791	ns	
				Tukey's	PCP+vehicle vs. PCP+PSEM308 (GABA-/c-fos+)	<0.0001	****	
				Tukey's	PCP+vehicle vs. METH+vehicle (GABA-/c-fos+)	0.4696	ns	
				Tukey's	PCP+vehicle vs. METH+PSEM308 (GABA-/c-fos+)	<0.0001	****	
				Tukey's	PCP+PSEM308 vs. METH+vehicle (GABA-/c-fos+)	<0.0001	****	

				Tukey's	PCP+PSEM308 vs. METH+PSEM308 (GABA-/c-fos+)	>0.9999	ns	
				Tukey's	METH+vehicle vs. METH+PSEM308 (GABA-/c-fos+)	<0.0001	****	
				Tukey's	saline+vehicle vs. saline+PSEM308 (GABA+/c-fos+)	>0.9999	ns	
				Tukey's	saline+vehicle vs. PCP+vehicle (GABA+/c-fos+)	0.9996	ns	
				Tukey's	saline+vehicle vs. PCP+PSEM308 (GABA+/c-fos+)	>0.9999	ns	
				Tukey's	saline+vehicle vs. METH+vehicle (GABA+/c-fos+)	0.9997	ns	
				Tukey's	saline+vehicle vs. METH+PSEM308 (GABA+/c-fos+)	>0.9999	ns	
				Tukey's	saline+PSEM308 vs. PCP+vehicle (GABA+/c-fos+)	0.9988	ns	
				Tukey's	saline+PSEM308 vs. PCP+PSEM308 (GABA+/c-fos+)	>0.9999	ns	
				Tukey's	saline+PSEM308 vs. METH+vehicle (GABA+/c-fos+)	0.9992	ns	
				Tukey's	saline+PSEM308 vs. METH+PSEM308 (GABA+/c-fos+)	>0.9999	ns	
				Tukey's	PCP+vehicle vs. PCP+PSEM308 (GABA+/c-fos+)	>0.9999	ns	
				Tukey's	PCP+vehicle vs. METH+vehicle (GABA+/c-fos+)	>0.9999	ns	
				Tukey's	PCP+vehicle vs. METH+PSEM308 (GABA+/c-fos+)	>0.9999	ns	
				Tukey's	PCP+PSEM308 vs. METH+vehicle (GABA+/c-fos+)	>0.9999	ns	
				Tukey's	PCP+PSEM308 vs. METH+PSEM308 (GABA+/c-fos+)	>0.9999	ns	
				Tukey's	METH+vehicle vs. METH+PSEM308 (GABA+/c-fos+)	>0.9999	ns	
**S15k**	Time to criterion in the NORT. Between treatment groups.	N=29 mice, 6-9/group	two-way ANOVA		Interaction	0.968	ns	F (1, 25) = 0.0016
					Row Factor (effect of drug treatment)	0.3082	ns	F (1, 25) = 1.082
					Column Factor (effect of chemogenetic treatment)	0.5467	ns	F (1, 25) = 0.3734
				Tukey's	saline+vehicle vs. saline+PSEM 308	0.971	ns	
				Tukey's	saline+vehicle vs. PCP+vehicle	0.8515	ns	
				Tukey's	saline+vehicle vs. PCP+PSEM 308	0.6571	ns	
				Tukey's	saline+PSEM 308 vs. PCP+vehicle	0.9898	ns	
				Tukey's	saline+PSEM 308 vs. PCP+PSEM 308	0.9058	ns	
				Tukey's	PCP+vehicle vs. PCP+PSEM 308	0.9737	ns	
**S15l**	Time to criterion in the NORT. Between treatment groups.	N=19 mice, 4-6/group	two-way ANOVA		Interaction	0.1629	ns	F (1, 15) = 2.154
					Row Factor (effect of drug treatment)	0.2032	ns	F (1, 15) = 1.771
					Column Factor (effect of chemogenetic treatment)	0.9297	ns	F (1, 15) = 0.008049
				Tukey's	saline+vehicle vs. saline+PSEM 308	0.7061	ns	
				Tukey's	saline+vehicle vs. METH +vehicle	0.1821	ns	
				Tukey's	saline+vehicle vs. METH +PSEM 308	0.7597	ns	
				Tukey's	saline+PSEM 308 vs. METH +vehicle	0.8076	ns	
				Tukey's	saline+PSEM 308 vs. METH +PSEM 308	0.9997	ns	
				Tukey's	METH +vehicle vs. METH +PSEM 308	0.7552	ns	
**S15m**	Total number of entries in the SAT. Between treatment groups.	N=31 mice, 6-9/group	two-way ANOVA	0	Interaction	0.2947	ns	F (1, 27) = 1.142
					Row Factor (effect of drug treatment)	0.6513	ns	F (1, 27) = 0.2089
					Column Factor (effect of chemogenetic treatment)	0.4889	ns	F (1, 27) = 0.4923
				Tukey's	saline+vehicle vs. saline+PSEM 308	0.9946	ns	
				Tukey's	saline+vehicle vs. PCP+vehicle	0.9677	ns	
				Tukey's	saline+vehicle vs. PCP+PSEM 308	0.8341	ns	
				Tukey's	saline+PSEM 308 vs. PCP+vehicle	0.9983	ns	
				Tukey's	saline+PSEM 308 vs. PCP+PSEM 308	0.7358	ns	
				Tukey's	PCP+vehicle vs. PCP+PSEM 308	0.5566	ns	
**S15n**	Total number of entries in the SAT. Between treatment groups.	N=19 mice, 4-5/group	two-way ANOVA		Interaction	0.0889	ns	F (1, 15) = 3.309
					Row Factor (effect of drug treatment)	0.7762	ns	F (1, 15) = 0.08375
					Column Factor (effect of chemogenetic treatment)	0.9676	ns	F (1, 15) = 0.001709
				Tukey's	saline+vehicle vs. saline+PSEM 308	0.5896	ns	
				Tukey's	saline+vehicle vs. METH +vehicle	0.6862	ns	
				Tukey's	saline+vehicle vs. METH +PSEM 308	0.9978	ns	
				Tukey's	saline+PSEM 308 vs. METH +vehicle	0.9957	ns	
				Tukey's	saline+PSEM 308 vs. METH +PSEM 308	0.4903	ns	
				Tukey's	METH +vehicle vs. METH +PSEM 308	0.5794	ns	

Supplementary table 22. Statistical details of Supplementary Fig. 16

Supplementary Fig.	Data/Comparison	N, n/group	Primary statistic	Post-hoc test	Comparison	p value	Notation	F/t statistic
S16b	Time to criterion in the NORT. Between treatment groups.	N=27 mice, 6-7/group	two-way ANOVA		Interaction	0.1019	ns	F (1, 23) = 2.902
					Row Factor (effect of PCP treatment)	0.7137	ns	F (1, 23) = 0.1379
					Column Factor (effect of clozapine treatment)	0.2307	ns	F (1, 23) = 1.516
				Tukey's	saline+vehicle vs. saline+clozapine	0.2046	ns	
				Tukey's	saline+vehicle vs. PCP+vehicle	0.7723	ns	
				Tukey's	saline+vehicle vs. PCP+clozapine	0.9244	ns	
				Tukey's	saline+clozapine vs. PCP+vehicle	0.6864	ns	
				Tukey's	saline+clozapine vs. PCP+clozapine	0.4891	ns	
				Tukey's	PCP+vehicle vs. PCP+clozapine	0.986	ns	
S16c	Total number of entries in the SAT. Between treatment groups.	N=27 mice, 6-7/group	two-way ANOVA		Interaction	0.1388	ns	F (1, 23) = 2.351
					Row Factor (effect of PCP treatment)	0.3201	ns	F (1, 23) = 1.033
					Column Factor (effect of clozapine treatment)	0.3614	ns	F (1, 23) = 0.8674
				Tukey's	saline+vehicle vs. saline+clozapine	0.3086	ns	
				Tukey's	saline+vehicle vs. PCP+vehicle	0.281	ns	
				Tukey's	saline+vehicle vs. PCP+clozapine	0.5417	ns	
				Tukey's	saline+clozapine vs. PCP+vehicle	>0.9999	ns	
				Tukey's	saline+clozapine vs. PCP+clozapine	0.9838	ns	
				Tukey's	PCP+vehicle vs. PCP+clozapine	0.9749	ns	
S16d	Total distance travelled at baseline. Between treatment groups.	N=31 mice, 7-8/group	two-way ANOVA		Interaction	0.1436	ns	F (1, 27) = 2.269
					Row Factor (effect of PCP treatment)	0.2853	ns	F (1, 27) = 1.188
					Column Factor (effect of clozapine treatment)	0.5969	ns	F (1, 27) = 0.2864
				Tukey's	saline+vehicle vs. saline+clozapine	0.4987	ns	
				Tukey's	saline+vehicle vs. PCP+vehicle	0.2929	ns	
				Tukey's	saline+vehicle vs. PCP+clozapine	0.6747	ns	
				Tukey's	saline+clozapine vs. PCP+vehicle	0.978	ns	
				Tukey's	saline+clozapine vs. PCP+clozapine	0.9904	ns	
				Tukey's	PCP+vehicle vs. PCP+clozapine	0.8966	ns	
S16g	Mean number of c-fos+ neurons 2 days after the end of treatment. Between treatment groups.	N=13 mice, 3-4/group	two-way ANOVA		Interaction	0.0007	***	F (1, 9) = 25.73
					Row Factor (effect of PCP treatment)	0.0016	**	F (1, 9) = 19.96
					Column Factor (effect of clozapine)	<0.0001	****	F (1, 9) = 45.16
				Tukey's	saline+vehicle vs. saline+clozapine	0.6827	ns	
				Tukey's	saline+vehicle vs. PCP+vehicle	0.0005	***	
				Tukey's	saline+vehicle vs. PCP+clozapine	0.4013	ns	
				Tukey's	saline+clozapine vs. PCP+vehicle	0.0001	***	
				Tukey's	saline+clozapine vs. PCP+clozapine	0.9693	ns	
				Tukey's	PCP+vehicle vs. PCP+clozapine	<0.0001	****	
S16h	Mean number of c-fos+/mCherry+ and cfos+/mCherry- neurons 2 days after the end of drug/saline injections. Between treatment groups.	N=13 mice, 3-4/group	two-way ANOVA		Interaction	0.0373	*	F (3, 18) = 3.490
					Row Factor (effect of treatment)	0.0005	***	F (3, 18) = 9.695
					Column Factor (mCherry+ or mCherry- cells)	<0.0001	****	F (1, 18) = 98.39
				Tukey's	saline+vehicle vs. saline+clozapine (mCherry+/c-fos+)	0.8155	ns	
				Tukey's	saline+vehicle vs. PCP+vehicle (mCherry+/c-fos+)	0.0044	**	
				Tukey's	saline+vehicle vs. PCP+clozapine (mCherry+/c-fos+)	0.508	ns	
				Tukey's	saline+clozapine vs. PCP+vehicle (mCherry+/c-fos+)	0.0007	***	
				Tukey's	saline+clozapine vs. PCP+clozapine (mCherry+/c-fos+)	0.9641	ns	
				Tukey's	PCP+vehicle vs. PCP+clozapine (mCherry+/c-fos+)	0.0001	***	
				Tukey's	saline+vehicle vs. saline+clozapine (mCherry-/c-fos+)	0.9982	ns	
				Tukey's	saline+vehicle vs. PCP+vehicle (mCherry-/c-fos+)	0.6731	ns	
				Tukey's	saline+vehicle vs. PCP+clozapine (mCherry-/c-fos+)	0.9991	ns	
				Tukey's	saline+clozapine vs. PCP+vehicle (mCherry-/c-fos+)	0.5719	ns	
				Tukey's	saline+clozapine vs. PCP+clozapine (mCherry-/c-fos+)	>0.9999	ns	
				Tukey's	PCP+vehicle vs. PCP+clozapine (mCherry-/c-fos+)	0.5448	ns	

Supplementary table 23. Statistical details of Supplementary Fig. 17

Supplementary Fig.	Data/Comparison	N, n/group	Primary statistic	Post-hoc test	Comparison	p value	Notation	F/t statistic
**S17b**	Mean number of c-fos+ neurons 2 days after the end of drug/saline injections. Between treatment groups.	N=15, 4-6/group	one-way ANOVA		Mean effect of grouyp	0.0009	***	F (2, 12) = 13.32
				Dunnett's	saline vs. PCP	0.0089	**	
				Dunnett's	saline vs. METH	0.0007	***	
**S17c**	Mean number of c-fos+ neurons 2 days after the end of drug/saline injections in mice sacrificed 11-to-12 hours after	N=10, 3-4/group	one-way ANOVA		Mean effect of grouyp	0.4123	***	F (2, 7) = 1.008
				Dunnett's	saline vs. PCP	0.8874	ns	
				Dunnett's	saline vs. METH	0.5686	ns	
**S17d**	Mean number of c-fos+/mCherry+ and cfos+/mCherry- neurons 2 days after the end of drug/saline injections. Between treatment groups.	N=15, 4-6/group	two-way ANOVA	Within each column, compare rows	Interaction	0.0057	**	F (2, 24) = 6.462
					Row Factor (effect of treatment)	<0.0001	****	F (2, 24) = 22.34
					Column Factor (mCherry+ or mCherry- cells)	<0.0001	****	F (1, 24) = 74.07
				Tukey's	saline vs. PCP (c-fos+/mCherry+)	0.0002	***	
				Tukey's	saline vs. METH (c-fos+/mCherry+)	<0.0001	****	
				Tukey's	PCP vs. METH (c-fos+/mCherry+)	0.0657	ns	
				Tukey's	saline vs. PCP (c-fos+/mCherry-)	0.2073	ns	
				Tukey's	saline vs. METH (c-fos+/mCherry-)	0.1367	ns	
				Tukey's	PCP vs. METH (c-fos+/mCherry-)	0.941	ns	
**S17e**	Mean fraction of PV+ neurons 2 days after the end of drug/saline injections. Between treatment groups.	N=12, 4/group	one-way ANOVA		Mean effect of group	0.8721	ns	F (2, 9) = 0.1389
				Dunnett's	saline vs. PCP	0.8541	ns	
				Dunnett's	saline vs. METH	0.8716	ns	

Supplementary table 24. Statistical details of Supplementary Fig. 18

Supplementary Fig.	Data/Comparison	N, n/group	Primary statistic	Post-hoc test	Comparison	p value	Notation	F/t statistic
S18c	Mean number of c-fos+ neurons 2 days after the end of treatment. Between treatment groups.	N=19 mice, 3-4/group	two-way ANOVA		Interaction	0.0003	***	F (2, 13) = 15.78
					Row Factor (effect of PCP/METH treatment)	<0.0001	****	F (2, 13) = 22.46
					Column Factor (effect of PSEM)	<0.0001	****	F (1, 13) = 92.90
				Tukey's	saline+vehicle vs. saline+PSEM308	0.707	ns	
				Tukey's	saline+vehicle vs. PCP+vehicle	<0.0001	****	
				Tukey's	saline+vehicle vs. PCP+PSEM308	0.8637	ns	
				Tukey's	saline+vehicle vs. METH+vehicle	0.0002	***	
				Tukey's	saline+vehicle vs. METH+PSEM308	>0.9999	ns	
				Tukey's	saline+PSEM308 vs. PCP+vehicle	<0.0001	****	
				Tukey's	saline+PSEM308 vs. PCP+PSEM308	0.9996	ns	
				Tukey's	saline+PSEM308 vs. METH+vehicle	<0.0001	****	
				Tukey's	saline+PSEM308 vs. METH+PSEM308	0.7309	ns	
				Tukey's	PCP+vehicle vs. PCP+PSEM308	<0.0001	****	
				Tukey's	PCP+vehicle vs. METH+vehicle	0.5394	ns	
				Tukey's	PCP+vehicle vs. METH+PSEM308	<0.0001	****	
				Tukey's	PCP+PSEM308 vs. METH+vehicle	<0.0001	****	
				Tukey's	PCP+PSEM308 vs. METH+PSEM308	0.8731	ns	
				Tukey's	METH+vehicle vs. METH+PSEM308	0.0004	***	
S18d	Mean number of c-fos+/GABA- and c-fos+/GABA+ neurons 2 days after the end of treatment. Between treatment groups.	N=19 mice, 3-4/group	two-way ANOVA		Interaction	<0.0001	****	F (5, 26) = 28.95
					Row Factor (effect of PCP/METH treatment)	<0.0001	****	F (5, 26) = 38.21
					Column Factor (effect of PSEM)	<0.0001	****	F (1, 26) = 1385
				Tukey's	saline+vehicle vs. saline+PSEM308 (c-fos+/GABA-)	0.2789	ns	
				Tukey's	saline+vehicle vs. PCP+vehicle (c-fos+/GABA-)	<0.0001	****	
				Tukey's	saline+vehicle vs. PCP+PSEM308 (c-fos+/GABA-)	0.284	ns	
				Tukey's	saline+vehicle vs. METH+vehicle (c-fos+/GABA-)	<0.0001	****	
				Tukey's	saline+vehicle vs. METH+PSEM308 (c-fos+/GABA-)	>0.9999	ns	
				Tukey's	saline+PSEM308 vs. PCP+vehicle (c-fos+/GABA-)	<0.0001	****	
				Tukey's	saline+PSEM308 vs. PCP+PSEM308 (c-fos+/GABA-)	>0.9999	ns	
				Tukey's	saline+PSEM308 vs. METH+vehicle (c-fos+/GABA-)	<0.0001	****	
				Tukey's	saline+PSEM308 vs. METH+PSEM308 (c-fos+/GABA-)	0.3894	ns	
				Tukey's	PCP+vehicle vs. PCP+PSEM308 (c-fos+/GABA-)	<0.0001	****	
				Tukey's	PCP+vehicle vs. METH+vehicle (c-fos+/GABA-)	0.8071	ns	
				Tukey's	PCP+vehicle vs. METH+PSEM308 (c-fos+/GABA-)	<0.0001	****	
				Tukey's	PCP+PSEM308 vs. METH+vehicle (c-fos+/GABA-)	<0.0001	****	
				Tukey's	PCP+PSEM308 vs. METH+PSEM308 (c-fos+/GABA-)	0.3952	ns	
				Tukey's	METH+vehicle vs. METH+PSEM308 (c-fos+/GABA-)	<0.0001	****	
				Tukey's	saline+vehicle vs. saline+PSEM308 (c-fos+/GABA+)	>0.9999	ns	
				Tukey's	saline+vehicle vs. PCP+vehicle (c-fos+/GABA+)	0.986	ns	
				Tukey's	saline+vehicle vs. PCP+PSEM308 (c-fos+/GABA+)	>0.9999	ns	
				Tukey's	saline+vehicle vs. METH+vehicle (c-fos+/GABA+)	0.8293	ns	
				Tukey's	saline+vehicle vs. METH+PSEM308 (c-fos+/GABA+)	0.9979	ns	
				Tukey's	saline+PSEM308 vs. PCP+vehicle (c-fos+/GABA+)	0.9924	ns	
				Tukey's	saline+PSEM308 vs. PCP+PSEM308 (c-fos+/GABA+)	>0.9999	ns	
				Tukey's	saline+PSEM308 vs. METH+vehicle (c-fos+/GABA+)	0.8809	ns	
				Tukey's	saline+PSEM308 vs. METH+PSEM308 (c-fos+/GABA+)	0.9991	ns	
				Tukey's	PCP+vehicle vs. PCP+PSEM308 (c-fos+/GABA+)	0.9859	ns	
				Tukey's	PCP+vehicle vs. METH+vehicle (c-fos+/GABA+)	0.9947	ns	

				Tukey's	PCP+vehicle vs. METH+PSEM308 (c-fos+/GABA+)	>0.9999	ns	
				Tukey's	PCP+PSEM308 vs. METH+vehicle (c-fos+/GABA+)	0.8457	ns	
				Tukey's	PCP+PSEM308 vs. METH+PSEM308 (c-fos+/GABA+)	0.9975	ns	
				Tukey's	METH+vehicle vs. METH+PSEM308 (c-fos+/GABA+)	0.9767	ns	
**S18f**	Total distance travelled at baseline. Between treatment groups.	N=25 mice, 4-8/group	two-way ANOVA		Interaction	0.5533	ns	F (1, 21) = 0.3629
					Row Factor (effect of drug treatment)	0.5748	ns	F (1, 21) = 0.3248
					Column Factor (effect of chemogenetic treatment)	0.4622	ns	F (1, 21) = 0.5609
				Tukey's	saline+vehicle vs. saline+PSEM308	0.8091	ns	
				Tukey's	saline+vehicle vs. PCP+vehicle	>0.9999	ns	
				Tukey's	saline+vehicle vs. PCP+PSEM308	0.9991	ns	
				Tukey's	saline+PSEM308 vs. PCP+vehicle	0.8096	ns	
				Tukey's	saline+PSEM308 vs. PCP+PSEM308	0.8751	ns	
				Tukey's	PCP+vehicle vs. PCP+PSEM308	0.9995	ns	
**S18g**	Time to criterion in the NORT. Between treatment groups.	N=23 mice, 4-7/group	two-way ANOVA		Interaction	0.8822	ns	F (1, 19) = 0.02257
					Row Factor (effect of drug treatment)	0.2993	ns	F (1, 19) = 1.139
					Column Factor (effect of chemogenetic treatment)	0.8633	ns	F (1, 19) = 0.03045
				Tukey's	saline+vehicle vs. saline+PSEM308	0.9964	ns	
				Tukey's	saline+vehicle vs. PCP+vehicle	0.7881	ns	
				Tukey's	saline+vehicle vs. PCP+PSEM308	0.7965	ns	
				Tukey's	saline+PSEM308 vs. PCP+vehicle	0.9287	ns	
				Tukey's	saline+PSEM308 vs. PCP+PSEM308	0.9292	ns	
				Tukey's	PCP+vehicle vs. PCP+PSEM308	>0.9999	ns	
**S18h**	Total number of entries in the SAT. Between treatment groups.	N=25 mice, 4-8/group	two-way ANOVA		Interaction	0.3372	ns	F (1, 21) = 0.9648
					Row Factor (effect of drug treatment)	0.4506	ns	F (1, 21) = 0.5911
					Column Factor (effect of chemogenetic treatment)	0.4776	ns	F (1, 21) = 0.5228
				Tukey's	saline+vehicle vs. saline+PSEM308	0.9983	ns	
				Tukey's	saline+vehicle vs. PCP+vehicle	0.9984	ns	
				Tukey's	saline+vehicle vs. PCP+PSEM308	0.6879	ns	
				Tukey's	saline+PSEM308 vs. PCP+vehicle	>0.9999	ns	
				Tukey's	saline+PSEM308 vs. PCP+PSEM308	0.6603	ns	
				Tukey's	PCP+vehicle vs. PCP+PSEM308	0.5351	ns	
**S18i**	Total distance travelled at baseline. Between treatment groups.	N=34 mice, 7-9/group	two-way ANOVA		Interaction	0.6981	ns	F (1, 30) = 0.1534
					Row Factor (effect of drug treatment)	0.1768	ns	F (1, 30) = 1.913
					Column Factor (effect of chemogenetic treatment)	0.1476	ns	F (1, 30) = 2.209
				Tukey's	saline+vehicle vs. saline+PSEM308	0.5789	ns	
				Tukey's	saline+vehicle vs. METH+vehicle	0.8861	ns	
				Tukey's	saline+vehicle vs. METH+PSEM308	0.9998	ns	
				Tukey's	saline+PSEM308 vs. METH+vehicle	0.2238	ns	
				Tukey's	saline+PSEM308 vs. METH+PSEM308	0.6223	ns	
				Tukey's	METH+vehicle vs. METH+PSEM308	0.8533	ns	
**S18j**	Time to criterion in the NORT. Between treatment groups.	N=32 mice, 7-9/group	two-way ANOVA		Interaction	0.7501	ns	F (1, 28) = 0.1034
					Row Factor (effect of drug treatment)	0.771	ns	F (1, 28) = 0.08636
					Column Factor (effect of chemogenetic treatment)	0.8832	ns	F (1, 28) = 0.02200
				Tukey's	saline+vehicle vs. saline+PSEM308	0.9882	ns	
				Tukey's	saline+vehicle vs. METH+vehicle	>0.9999	ns	
				Tukey's	saline+vehicle vs. METH+PSEM308	0.9996	ns	
				Tukey's	saline+PSEM308 vs. METH+vehicle	0.9893	ns	
				Tukey's	saline+PSEM308 vs. METH+PSEM308	0.9742	ns	
				Tukey's	METH+vehicle vs. METH+PSEM308	0.9993	ns	
**S18k**	Total number of entries in the SAT. Between treatment groups.	N=34 mice, 8-9/group	two-way ANOVA		Interaction	0.9063	ns	F (1, 30) = 0.01409
					Row Factor (effect of drug treatment)	0.771	ns	F (1, 30) = 0.08623
					Column Factor (effect of chemogenetic treatment)	0.1188	ns	F (1, 30) = 2.578
				Tukey's	saline+vehicle vs. saline+PSEM308	0.6197	ns	
				Tukey's	saline+vehicle vs. METH+vehicle	0.9912	ns	
				Tukey's	saline+vehicle vs. METH+PSEM308	0.5186	ns	
				Tukey's	saline+PSEM308 vs. METH+vehicle	0.804	ns	
				Tukey's	saline+PSEM308 vs. METH+PSEM308	0.9993	ns	
				Tukey's	METH+vehicle vs. METH+PSEM308	0.7209	ns	
